# Association of Novel *Streptococcus sanguinis* Virulence Factors With Pathogenesis in a Native Valve Infective Endocarditis Model

**DOI:** 10.3389/fmicb.2020.00010

**Published:** 2020-01-31

**Authors:** Anthony M. Martini, Bridget S. Moricz, Allison K. Ripperger, Phuong M. Tran, Molly E. Sharp, Ana N. Forsythe, Katarina Kulhankova, Wilmara Salgado-Pabón, Bradley D. Jones

**Affiliations:** ^1^Department of Microbiology & Immunology, The Roy J. and Lucille A. Carver College of Medicine, The University of Iowa, Iowa City, IA, United States; ^2^Graduate Program in Genetics, The University of Iowa, Iowa City, IA, United States

**Keywords:** streptococcus, sanguinis, commensal, endocarditis, pathogenesis, heart, valve

## Abstract

*Streptococcus sanguinis* (*S. sanguinis*) is an abundant oral commensal which can cause disseminated human infection if it gains access to the bloodstream. The most important among these diseases is infective endocarditis (IE). While virulence phenotypes of *S. sanguinis* have been correlated to disease severity, genetic factors mediating these phenotypes, and contributing to pathogenesis are largely uncharacterized. In this report, we investigate the roles of 128 genes in virulence-related phenotypes of *S. sanguinis* and characterize the pathogenic potential of two selected mutants in a left-sided, native valve IE rabbit model. Assays determining the ability of our mutant strains to produce a biofilm, bind to and aggregate platelets, and adhere to or invade endothelial cells identified sixteen genes with novel association to these phenotypes. These results suggest the presence of many uncharacterized genes involved in IE pathogenesis which may be relevant for disease progression. Two mutants identified by the above screening process – *SSA_1099*, encoding an RTX-like protein, and *mur2*, encoding a peptidoglycan hydrolase – were subsequently evaluated *in vivo*. Wild type (WT) *S. sanguinis* reliably induced cardiac vegetations, while the *SSA_1099* and *mur2* mutants produced either no vegetation or vegetations of small size. Splenomegaly was reduced in both mutant strains compared to WT, while pathology of other distal organs was indistinguishable. Histopathology analyses suggest the cardiac lesions and vegetations in this model resemble those observed in humans. These data indicate that *SSA_1099* and *mur2* encode virulence factors in *S. sanguinis* which are integral to pathogenesis of IE.

## Introduction

*Streptococcus sanguinis* (*S. sanguinis*) is a commensal organism important in promoting oral health but may become pathogenic if given the opportunity. The oral cavity of humans is a rich environment providing a moist, warm niche for colonization by hundreds of different bacterial species that are typically harmless or beneficial to the oral health of humans. The most abundant of these bacterial species are those belonging to the genus Streptococcus. Within this environment, *S. sanguinis*, a primary colonizer of the tooth surface, provides benefits to the human host by protecting against the deleterious effects of another microorganism, *S. mutans*, the pathogen responsible for tooth decay and caries (Becker et al., 2002; Kreth et al., 2008). Oral streptococci often cause disease when the organisms move from the mouth into the human bloodstream. Of importance is infective endocarditis (IE), which is an infection of the heart valves and/or endocardium, because the infection is associated with complications that include congestive heart failure, aneurysm and stroke (Mylonakis and Calderwood, 2001; Murdoch et al., 2009; DeSimone et al., 2015). Despite improved diagnostic and treatment options, studies continue to report that endocarditis mortality rates range from 12 to 45% (Thuny et al., 2012; Bor et al., 2013).

Infective endocarditis occurs when microorganisms enter the bloodstream and colonize the cardiac endothelium. Oral streptococci are responsible for ca. 20% of the global IE burden, with greater incidence in developing and resource-limited regions (Yew and Murdoch, 2012; Vogkou et al., 2016). Among these species, *S. sanguinis* is particularly important as it is one of the most frequent causative agents of IE, comprising between 18 and 30% of cases (Horaud and Delbos, 1984; Douglas et al., 1993; Filippo et al., 2006). Bacteria in the oral cavity are frequent sources of transient bacteremia due to dental procedures as well as normal oral care such as brushing and mastication, which can provide oral bacteria access to the bloodstream. In addition, poor oral health can create an environment of intermittent transient bacteremia due to inflammation and/or more severe damage oral epithelium. It is believed that an essential step in the initiation of IE is adherence of *S. sanguinis* to circulating platelets in the bloodstream and/or binding to submucosal proteins such as collagen at regions of endothelial disruption or damage (Keynan and Rubinstein, 2013; Holland et al., 2016). Due to the seriousness of endocarditis and the impracticality of long-term antibiotic usage for transient bloodstream infections, understanding the virulence mechanisms of *S. sanguinis* is of high value. Improved understanding of the virulence factors and mechanisms of this organism will allow the development of customized therapies for treatment and prevention of bacterial endocarditis caused by *S. sanguinis*.

Microorganisms capable of causing IE must successfully navigate several stages of disease progression and host challenges to establish a cardiac infection (Werdan et al., 2014). Principally, this involves (i) entrance to and survival within the bloodstream, (ii) adherence via host ECM components and/or platelets at the site of valve damage, (iii) colonization of the valvular endothelium, and (iv) development of and growth within the vegetation. The need to respond to these different conditions indicate this organism has distinct mechanisms for growing and survival in the mouth and causing endocarditis in the bloodstream. Some metabolic and virulence genes involved in these processes have been characterized. The ability of this organism to form a biofilm, as part of its colonization strategy in the mouth, relies both on its ability to synthesize adhesive glucans from sucrose and to induce the release of extracellular DNA (Ge et al., 2008; Zhu et al., 2018), which plays an important role in biofilm formation on the surfaces of teeth. In contrast, cardiac vegetations formed by colonization of the endothelium are expected to be composed of human platelets, fibrin and bacteria (Jung et al., 2012; Mancini et al., 2016), though glucans and extracellular DNA have also been reported to retain their importance in this environment (Jung et al., 2015, 2017). To form this specialized platelet-containing biofilm *S. sanguinis* must express virulence factors that function within the context of heart tissue colonization and growth. A prior investigation using signature-tagged mutagenesis evaluated 800 mutants *in vivo* and demonstrated significantly attenuated virulence of strains containing mutations in *bacA*, *thrB*, *nrdD*, and *purB*, which were determined to be necessary for growth and survival in different host environments (Paik et al., 2005). Similarly, mutations in the *nrdHEKF* genes encoding the aerobic ribonucleotide reductase system abrogated virulence *in vivo* by compromising its ability to grow in blood, which contains between 6 to 12% O_2_ (Rhodes et al., 2014). Other described virulence genes include *srtA*, which encodes a sortase required for anchoring proteins to the cell wall and is important for *S. sanguinis* adherence and colonization (Yamaguchi et al., 2006); *srpA*, encoding a serine-rich repeat adhesin that binds with high affinity to human platelets in whole blood and may facilitate colonization of the endocardium by “riding” platelets to the site of damage (Plummer et al., 2005; Deng et al., 2014; Bensing et al., 2016); and *nt5e*, an ecto-5′-nucleotidase involved in virulence and platelet aggregation which may also function as an immunosuppressant through the generation of adenosine (Fan et al., 2012).

In this report, we have used a bioinformatics approach to construct a large set of *S. sanguinis* SK36 mutants (128 mutants) to be examined for potential involvement in the virulence mechanisms of *S. sanguinis* endocarditis. We have compared the phenotypes of these mutant strains to the isogenic parent *S. sanguinis* SK36 using *in vitro* virulence assays to provide a more comprehensive picture of the factors that *S. sanguinis* employs to cause heart infections. These experiments identified 16 new genetic loci that appear to be important in platelet interactions or adherence/invasion interactions with host cells. We also examined the ability of two of these mutants to cause vegetations in a rabbit model that simulates left-sided, native valve endocarditis. The results obtained from these experiments are presented here in detail.

## Materials and Methods

### Bacterial Strains and Growth Conditions

Mutations in putative virulence genes are listed in Supplementary Table S1. WT *S. sanguinis* strain SK36, a human dental plaque isolate, was generously provided by Dr. Mark Herzberg. Routine culturing was performed at 37°C under stationary conditions in brain heart infusion broth (BHI; research products international (RPI), Mt. Prospect, IL) or on plates (BHA) supplemented with 1.5% agar (BD; Becton, Dickinson and Company, Sparks, MD, United States) in an atmosphere containing 5% CO_2_. Growth curves were performed in 100 μL aliquots of Todd-Hewitt broth (TH; Dot Scientific) supplemented with 0.3% yeast extract (Difco). Where indicated, mutations in *S. sanguinis* SK36 genes were selected using kanamycin (RPI) at a final concentration of 500 μg/ml.

For investigations of biofilm production, we utilized a modification of the chemically defined medium described previously (Loo et al., 2000). This biofilm medium contains 1.0% sucrose, 0.8% glucose, 0.2% Casamino acids, 58 mM K_2_HPO_4_, 15 mM KH_2_PO_4_, 10 mM (NH_4_)_2_SO_4_, and 35 mM NaCl. The following stock solutions were supplied at the indicated concentrations: MgSO_4_ [2 mM], MnCl_4_ [0.1 mM], L-arginine [1 mM], L-glutamic acid [4 mM], L-tryptophan [0.1 mM], L-cysteine [1.3 mM], biotin [0.05 μM], nicotinic acid [0.04 mM], D-pantothenic acid [0.01 mM], pyridoxamine [0.1 mM], thiamine [0.3 μM], and riboflavin [1 μM]. The complete medium was filter-sterilized and could be stored at 4°C for approximately 1 week.

### Construction of Non-polar Mutants

Mutagenesis of *S. sanguinis* SK36 to create marked gene deletion strains was performed using previously reported primers and methods (Xu et al., 2011). Briefly, ca. 1 kb regions upstream and downstream of the target gene were separately amplified via polymerase chain reaction (PCR). The internal primers for these reactions were modified to contain 5′ regions complementary to the neomycin phosphotransferase II (*neo*) gene from plasmid pKD4 (Datsenko and Wanner, 2000). Individual PCRs were performed using the following conditions: 98°C for 30 s; 30 cycles of 98°C for 10 s; 55°C for 30 s; 72°C for 30 s; and 72°C for 5 m. Equal concentrations of upstream, downstream, and *neo* fragments were combined and amplified by splicing overlap extension (SOE) PCR to produce a linear upstream-*neo*-downstream linear fragment. The SOE PCR conditions were as follows: 98°C for 30 s; 30 cycles of 98°C for 10 s; 65°C for 30 s; 72°C for 2 m; and 72°C for 5 m. Phusion DNA polymerase (NEB) was used for all reactions. All PCR fragments and constructs were purified using a QIAquick PCR Purification Kit (Qiagen) prior to downstream applications.

Transformation of *S. sanguinis* SK36 with the linear DNA constructs generated above was performed as described previously (Xu et al., 2011). Overnight cultures of *S. sanguinis* SK36 were diluted 1:10 into 1 mL of fresh BHI supplemented with 0.4% bovine serum albumin (BSA) and statically incubated at 37°C in a candle jar until an OD_660_ of 0.2–0.3 was reached. Subsequently, ca. 100–200 ng of SOE PCR construct and 10 μg/mL of competence stimulating peptide (CSP; GenScript) were added to the bacterial suspension and the transformation mixture was incubated for 2 h before plating on BHA Kan^500^. Plates were incubated at 37°C in 5% CO_2_ for 48 h to select for mutant strains. Successful integration was determined by PCR amplification using the external primers and comparison of the fragment size generated from a mutant to WT by gel electrophoresis. Flanking regions from *in vivo* tested strains SK36 Δ*mur2* and Δ*SSA_1099* were each PCR amplified twice in independent reactions and sequenced by the method of Sanger.

### Biofilm Production

Assessment of biofilm production by the *S. sanguinis* mutant strains was performed *in vitro* using a polystyrene microtiter plate assay (Costar 3596; Corning Incorporated, Corning, NY, United States) according to published methods (Ge et al., 2008) with some modification. Overnight cultures of *S. sanguinis* cultured in BHI broth under static conditions in 5% CO_2_ were washed once in PBS and resuspended to an OD_600_ of 1.0. Microfuge tubes containing 1 mL of biofilm media were inoculated 1:100 with the appropriate bacterial strain, mixed thoroughly, and 100 μL of solution aliquoted in quadruplicate to the wells of a 96-well microtiter plate. Plates were sealed with Parafilm to minimize evaporation and incubated for 24 h in a 37°C anaerobic chamber (Coy Laboratory Products) with an atmosphere of 10% H_2_, 5% CO_2_, and 85% N_2_. Bacterial growth was determined by measuring the OD_450_ after 24 h using a microtiter plate reader (Tecan; Infinite M200 PRO). Growth medium was decanted, and the wells washed three times by submersion in distilled water to remove non-adherent cells. Adherent cells were heat-fixed by incubation at 60°C for 45 min. To stain adherent biomass, wells were incubated for 15 min with 0.1% crystal violet, after which the stain was decanted, and wells washed three times as before. Plates were inverted and allowed to dry for 5 min before destaining with 150 μL of 40 mM HCl in 95% ethanol. After 30 min of stationary incubation on the benchtop, 100 μL of the destain solution was transferred to a fresh microtiter plate and the solubilized crystal violet quantified at an absorbance of 590 nm using an Infinite 200PRO plate reader (Tecan).

### Platelet Adherence

Platelets were isolated by centrifugation at 800 rcf for 15 min, resuspended in one-tenth volume citrate buffer (pH 6.5), and washed three times as above. Platelets were concentrated further by resuspension in a one-tenth volume of Tyrode’s modified salt solution (Tyrode’s buffer; pH 7.4), enumerated using a hemocytometer, and adjusted to the proper concentration using Tyrode’s. Adherence of human platelets to bacteria was performed as described previously (Kerrigan et al., 2002). Briefly, overnight cultures of *S. sanguinis* were washed and resuspended in PBS to ca. 2.5 × 10^8^ CFU/ml. 100 μL aliquots (∼2.5 × 10^7^ CFU/well) were distributed to wells of a 96-well microtiter plate in triplicate and incubated for 2 h at 37°C to permit bacterial adherence. Inoculated wells were then washed and blocked with 1% BSA in PBS for 1 h at 37°C. Tyrode’s buffer was used to wash the wells before addition of ca. 2.5 × 10^7^ platelets, at which point plates were incubated for 30 min at 37°C. Non-adherent platelets were removed by washing three times with Tyrode’s buffer and bacteria-bound platelets were lysed by the addition of 100 μL lysis buffer (0.1 M sodium acetate, 0.1% Triton X-100) containing 10 mM *p-*nitrophenylphosphate (pNPP). Plates were incubated for 30 min at 37°C and reactions stopped by the addition of 100 μL 1 M NaOH. Production of yellow nitrophenol was quantified at an absorbance of 410 nm using a plate reader.

### Light Transmission Aggregometry

A microtiter plate assay was adapted to determine the aggregation of platelets in platelet-rich plasma (PRP) for the analysis of bacterial agonists (Vinholt et al., 2017). Heparinized whole blood was obtained from a single donor to minimize individual variability. PRP was generated by centrifugation at 200 rcf for 10 min, and platelet-poor plasma (PPP) was generated by centrifugation of the remaining sample at 2,000 rcf for 20 min. Platelets were enumerated via 100-fold dilution in 1% ammonium oxalate and adjusted to a concentration of 3–4 × 10^8^/mL using autologous PPP. Concurrently, overnight cultures of *S. sanguinis* were pelleted and the supernatant removed by aspiration. Cell pellets were resuspended in filter-sterilized isotonic glucose and normalized to an OD_600_ of 10.0 in the same solution. *S. sanguinis* suspensions were distributed in 10 μL aliquots to a 96-well microtiter plate in triplicate. Solutions of isotonic glucose alone or containing 0.5 μg/mL collagen type I (CHRONO-LOG; Havertown, PA, United States) were similarly distributed as negative and positive controls, respectively. Once the plate reader reached 37°C, 90 μL of either PRP or PPP was rapidly added to one concomitant half of each experimental and control condition. Platelet aggregation was monitored by reading the OD_595_ immediately after addition of PRP or PPP and every 150 s thereafter. The reaction was allowed to proceed for 40 min. Total aggregation was determined by subtracting the basal aggregation rate observed at each time point in a PRP-only control. The percent aggregation over time for each strain was calculated via the following formula:

$$\frac{I\,n\,i\,t\,i\,a\,l\,P\,R\,P\,S\,a\,m\,p\,l\,e-P\,R\,P\,S\,a\,m\,p\,l\,e\,T\,i\,m\,e\,p\,o\,i\,n\,t}{I\,n\,i\,t\,i\,a\,l\,P\,R\,P\,S\,a\,m\,p\,l\,e}*100$$

### Tissue Culture

Adherence and invasion assays of immortalized human epithelial (HeLa) and aortic endothelial (HAEC) cells (Herrera et al., 2017) were performed largely as previously described (Okahashi et al., 2010). HeLa and HAEC cells were cultured in RPMI 1640 containing 10% fetal bovine serum (FBS) and Medium 200 containing 10% low serum growth supplement (LSGS), respectively. Approximately 5 × 10^4^ of either cell type was seeded to 24-well tissue culture plates in triplicate and incubated for 24 (HeLa cells) or 48 (HAEC cells) hours at 37°C with 5% CO_2_. Overnight cultures of *S. sanguinis* strains were washed once with PBS and added to each well at a multiplicity of infection (MOI) of 50 and incubated for an additional 3 h. To quantitate bacterial adherence, cells were washed three times with PBS and then fixed by the addition of 4% paraformaldehyde. Giemsa staining was performed for 20 min (HeLa) or 30 (HAEC) minutes and adherent bacteria enumerated using light microscopy. Bacterial cells were counted individually when present as part of a chain. Preliminary experiments performed on the impact of internalized bacteria on these counts indicated their effect was trivial, likely due to the >log difference between the two niches (data not shown). To quantify invasive bacteria, cells were washed as above and incubated for 1 h in cell-specific medium containing 100 μg/mL gentamicin. Cells were washed three times prior to lysis. HAECs were first incubated in 200 μL of 0.025% Trypsin-EDTA for 5 min at RT and then lysed by addition of 800 μL 1% Tween 20 in PBS for 20 min at 37°C. Lysis of HeLa cells was achieved by the addition of 500 μL of 0.1% Triton X-100 in PBS for 5 min at RT. Cell lysates were vigorously mixed by pipetting and dilutions of cell lysates plated on BHA to enumerate CFUs.

### Experimental Infective Endocarditis Model

The ability of *S. sanguinis* strains to induce IE was tested in a rabbit model of aortic injury. Induction of aortic injury was performed using healthy, outbred New Zealand White (NZW) rabbits (2–3 kg; Bakkom Rabbitry, Red Wing, MN) as previously described (Salgado-Pabón and Schlievert, 2016). Briefly, following exposure of the carotid artery of rabbits under anesthesia, a sterile catheter was extended toward the aorta through the left carotid artery and allowed to mechanically abrade the valve for 2 h. Catheters were then removed, and the carotid artery and neck incision closed. Bacteremia was initiated by intravenous injection through the marginal ear vein with ca. 1 –3 × 10^8^ CFU *S. sanguinis* resuspended in hospital-grade saline. This bacterial concentration has previously been demonstrated to induce cardiac vegetations in a native valve model of endocarditis using NZW rabbits (Fan et al., 2012). Infection was permitted to proceed for 7 days; general health and behavior of the rabbits was observed four times daily during the course of each experiment. After 7 days, animals were euthanized and necropsied to evaluate vegetation development and disseminated disease pathology.

Blood samples (ca. 3 mL) were collected at catheterization and immediately prior to euthanasia using syringes preloaded with heparin (10 USP/mL). Determinations of bacteremia following death or prior to euthanasia were performed by plating blood and dilutions thereof directly on BHA. To generate plasma, heparinized blood was centrifuged for 10 min at 5,000 rcf and the plasma transferred to a fresh microfuge tube. Samples were frozen at −80°C for future evaluation. Cardiac vegetations were excised from the heart valve of each rabbit, weighed, homogenized in 1 mL of sterile PBS using a Bio-Gen PRO200 homogenizer (PRO Scientific), and dilutions plated to determine the CFU present within each vegetation. In instances where no vegetation could be observed, the entirety of the valve was vigorously scraped with a sterile dissecting blade and vortexed in PBS for plating. In the absence of a vegetative mass, samples were not homogenized. Peripheral organs including the kidneys, spleen, liver, and lungs were removed and examined for gross pathology. Scoring of gross pathology was performed by three blinded investigators according to a scale developed by the Salgado-Pabón laboratory (Kulhankova et al., unpublished). Sections of each organ were preserved in 10% normal-buffered formalin (NBF).

### Statistical Analysis

Statistical analysis of data was performed with GraphPad Prism 8 (GraphPad Software, San Diego, CA, United States) using one-way analysis of variance (ANOVA), two-way ANOVA, Area Under the Curve, and linear regression. A *p* < 0.05 was considered significant for statistical hypothesis testing.

### Ethics Statement

All animal experiments were performed in accordance with the protocols of the Institutional Animal Care and Use Committee of the University of Iowa (protocol #1106138). The University of Iowa is a registered research facility with the U.S. Department of Agriculture (USDA) and complies with the Animal Welfare Act and Regulations (AWA/AWR). The facility holds an Animal Welfare Assurance through the Office of Laboratory Animal Welfare (OLAW) and complies with PHS Policy. Additionally, the facility is accredited through the Association for Assessment and Accreditation of Laboratory Animal Care International (AAALACi) and is committed to comply with the Guide for the Care and Use of Laboratory Animals.

Human platelets were acquired from the University of Iowa DeGowin Blood Center as PRP. We have received approval from the University of Iowa Institutional Review Board to use human blood components under protocol #201902770. All samples used for this work were anonymized. The patients/participants provided their written informed consent to participate in this study. All participants were over 16 years of age.

## Results

### *Streptococcus sanguinis* Mutant Library Construction

Bioinformatic analysis of the *S. sanguinis* SK36 genome was performed to identify genes with putative virulence functions including adherence, interactions with host cells including platelets and outer membrane proteins of unknown functions. This analysis led to the identification of 128 genes that were selected for mutagenesis and future study. The first step in mutant construction was amplification of a kanamycin-resistance gene fragment by PCR that was flanked by 1,000 bp upstream and downstream of the open reading frame. This linear DNA fragment was introduced into *S. sanguinis* SK36 by transformation and mutants in the gene of interest was selected by plating the transformation mix on BHA Kan^500^. Since a double crossover event was required to obtain colonies transformed with linear DNA, the kanamycin selection yielded mutants in a single step. To confirm that colonies had the correct genotype, confirmatory PCR was performed using a primer specific to the kanamycin resistance gene and a chromosomal primer outside the region present on the original mutagenic fragment. Following confirmation that a desired mutant was constructed, the strain was grown up and stored at −80°C for future experimentation.

### Microtiter Biofilm Formation

**FIGURE 1:**
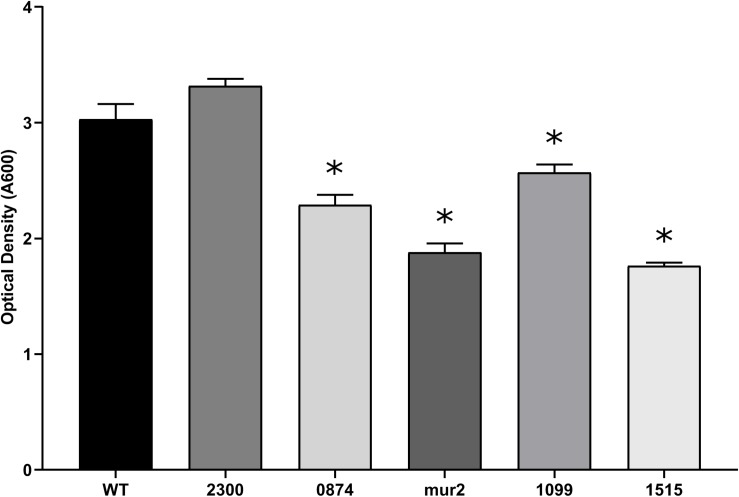
Putative virulence factors of *S. sanguinis* are associated with a reduction in biofilm production. Biofilm production of the indicated strains after 24 h of anaerobic growth in BM containing 1% sucrose. Inoculums of each strain were normalized after overnight growth in BHI. All strains exhibited growth in BM indistinguishable from WT (Supplementary Figure S1). Adherent biomass was quantified by retention of crystal violet and measuring the optical density after solubilization at an absorbance of 590 nm. Assays were performed in quadruplicate and three experiments were performed. Statistical analysis was performed using one-way ANOVA and corrected for multiple comparisons against the WT mean using Dunnett’s test. ^∗^*p* < 0.05. Error bars represent the standard error.

Cardiac vegetations, which develop over the course of IE, are considered biofilm-like structures composed of both bacterial and host factors (Elgharably et al., 2016). While *in vitro* biofilm formation on a plastic surface does not strongly correlate with *in vivo* virulence (Ge et al., 2008; Leuck et al., 2014), production of an extracellular matrix has been described to occur within streptococcal vegetations (Mills et al., 1984) and production of extracellular polysaccharide (EPS) correlates with streptococcal IE (Mills et al., 1984; Dall and Herndon, 1990). Consistent with a previous report (Ge et al., 2008), supplementation with the same concentration of glucose, a more physiologically relevant sugar, did not produce strong biofilms in this assay (data not shown). Therefore, we screened our mutant library for deficiencies in biofilm formation using sucrose as a stimulant of glucan production in a minimal biofilm medium. Mutants exhibiting deficiencies in adherent biomass accumulation, while retaining similar growth kinetics compared to WT, were identified by a reduction in crystal violet retention. We observed 81 of the 128 tested mutants to exhibit significant growth defects in minimal biofilm media compared to WT, and these strains were therefore not analyzed further for biofilm production (data not shown). Of the remaining 47 strains, we identified four mutants that exhibited reproducible deficiencies in adherent biomass (Figure 1) and that had growth indistinguishable from WT (Supplementary Figure S1). Strains Δ*mur2::kan* and Δ*SSA_1515::kan*, respectively, produced biofilms exhibiting ca. 62 and 58% (a ∼1.6-fold reduction) of the biomass produced by WT, the greatest decrease of any mutant strain observed under these conditions. *mur2* encodes a predicted peptidoglycan (PG) hydrolase with homology to the *mur2* gene identified in *S. iniae*, while *SSA_1515* encodes a hypothetical protein predicted to be involved in cell wall metabolism. Strain Δ*SSA_0874::kan* exhibited mean biomass accumulation ca. 75% (∼1.3-fold reduction) that of WT and is predicted to encode a protein with similarities to metabolite efflux functions. Δ*SSA_1099::kan* exhibited a modest decrease in mean biomass of ca. 15% (∼1.2-fold reduction) compared to WT. This gene encodes a predicted RTX-like protein. The Δ*SSA_2300::kan* strain is included to contrast mutants exhibiting a biofilm-deficient phenotype with one which does not.

### Platelet Interactivity

**FIGURE 2:**
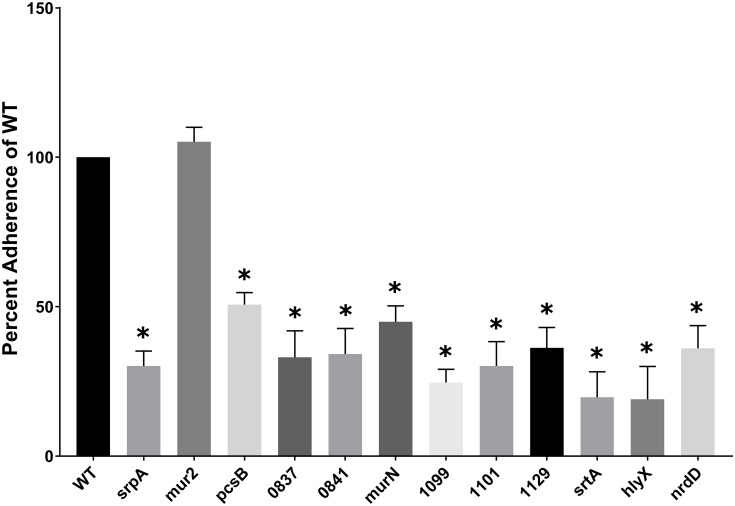
*S. sanguinis* mutants with a deficiency in human platelet binding. Adherence of purified platelets to immobilized bacteria. Microtiter wells were coated with a normalized concentration of bacterial cells and then blocked with BSA. Platelets resuspended in Tyrode’s buffer were added to the wells and incubated at 37°C to allow adhesion. Bound platelets were lysed in the presence of pNPP and the optical density at 410 nm measured. The ability of each strain to bind platelets was indirectly determined by quantitation of platelet acid phosphatase activity as measured by nitrophenol production from pNPP. Mutant strain platelet binding was compared to WT. Assays were performed in triplicate and three experiments were performed. Statistical analysis was performed using one-way ANOVA and corrected for multiple comparisons against the WT mean using Dunnett’s test. ^∗^*p* < 0.05. Error bars represent the standard error.

*S. sanguinis* interactions (adhesion, aggregation and/or activation) with platelets are known to correlate with IE disease progression (Herzberg et al., 1990, 1992, 2005), suggesting bacterial factors mediating these interactions are integral to IE pathogenesis. We compared the ability of purified human platelets to bind to *S. sanguinis* mutants with WT *S. sanguinis* SK36 using an *in vitro* pNPP phosphatase assay. As a consideration, we examined the ability of each *S. sanguinis* mutant strain to bind to the plastic of the microtiter plates, but we did not observe any differences (data not shown). Nine genes from our library with previously uncharacterized platelet interactivity associations were determined to influence binding to human platelets (Figure 2). The identified genes possess a range of known or predicted functions: *SSA_0019* encodes PG hydrolase, *pcsB*; *SSA_0837* encodes a predicted glycosyltransferase; the *SSA_0841* gene is predicted to encode a hypothetical protein with homology to accessory secretory protein (*asp5*), which is related to the platelet-binding glycoprotein *srpA* (Xu et al., 2007); the PG cross-linking protein, *murN*, is encoded by *SSA_0861*; *SSA_1099*, the RTX-like protein identified above, and *SSA_1101* are arranged in an apparent operon. *SSA_1101* is predicted to function as a membrane fusion protein in the transport system for the RTX adhesin encoded by *SSA_1099*; the *SSA_1129* gene product is a predicted iron transport lipoprotein associated with the twin-arginine translocation (TAT) pathway; a predicted hemolysin (*hlyX*) is encoded by *SSA_1761*; and *SSA_2230* encodes the anaerobic ribonucleoside triphosphate reductase, *nrdD*.

Most mutants identified were decreased for mean platelet adherence by ca. three- to fourfold (*nrdD*, *SSA_0837*, *0841*, *1099*, *1101*, *1129*), but ranged from ca. twofold (*pcsB* and *murN*) to fivefold (*hlyX*). The greatest decrease, which was observed for *hlyX*, exhibited compromised platelet binding ability like *srtA* (Figure 2). The platelet-binding glycoprotein, *srpA*, and *mur2* serve as positive (decreased binding) and negative (unaffected binding) controls, respectively. The ability to induce platelet aggregation has previously been demonstrated to correlate with the ability of *S. sanguinis* strains to cause experimental IE. Although platelet adherence and aggregation are often associated, these phenotypes can be decoupled in *S. sanguinis* (Herzberg et al., 1990). Thus, we endeavored to determine whether the mutants deficient for platelet adherence were simultaneously compromised in their ability to induce platelet aggregation. Platelet aggregation was determined by monitoring the turbidity of a platelet-bacterial suspension over time at OD_595_ and compared to time zero. The decrease in OD_595_ observed from a PRP-only control was subtracted from each experimental time point to account for the basal aggregation of PRP under these conditions. We define maximum platelet aggregation as the time point at which WT SK36 exhibited the greatest percent aggregation.

**FIGURE 3:**
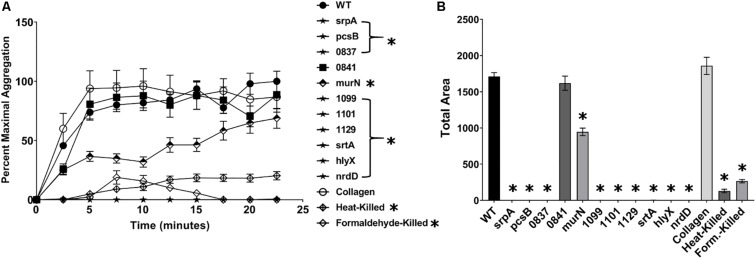
*S. sanguinis* mutants exhibiting a reduction in platelet binding are deficient in aggregating platelets. Ability of mutant strains to induce platelet aggregation in platelet-rich plasma (PRP). The concentration of bacterial strains was normalized to an OD_600_ of 10, and 10 μL of each culture was added to the wells of a microtiter plate. 90 μL of PRP was quickly added to the bacterial solution and the microtiter plate transferred to a pre-warmed plate reader, where the initial OD at 595 nm was measured. The plate reader was set to shake constantly and read the OD every 2.5 min. **(A)** Platelet aggregation was determined by the decrease in OD over time and set relative to the maximum aggregation observed in WT. Strains labeled with stars and in brackets were indistinguishable from baseline. **(B)** Total area of platelet aggregation over the experimental time course. Assays were performed in triplicate and three experiments were performed. Statistical analysis was performed using two-way ANOVA for the time course and one-way ANOVA for Area Under Curve, both corrected for multiple comparisons against the WT mean using Dunnett’s test. ^∗^*p* < 0.05. Error bars represent the standard error.

Of the mutants identified as deficient in adherence to human platelets, all but one (*SSA_0841*) exhibited a decreased capacity to induce platelet aggregation in PRP (Figures 3A,B). Collagen (type I) – a known platelet agonist – served as a positive control. Mutant strains *pcsB*, *srpA*, *SSA_0837*, *SSA_1099*, *SSA_1101*, *SSA_1129*, *srtA*, *hlyX*, and *nrdD* exhibited a complete, or nearly complete, loss of platelet aggregation activity compared to WT, and this was evident at both early and late time points (Figure 3A). In contrast, we observed an intermediate, delayed phenotype associated with the Δ*murN* mutant. Total aggregation was ca. half that of WT at over the course of the experiment (Figure 3B). We did not observe significant increases in aggregation for *murN*, or any other strain, over longer time frames (data not shown). Formaldehyde- and heat-killed bacteria were used as controls.

### Adherence and Invasion to Human Cells

To establish and maintain a cardiac infection, blood-borne bacteria must adhere to and subsequently colonize human tissues, either directly or indirectly via extracellular matrix molecules. Invasion of the endocardium has also been suggested to mediate recalcitrant or recurrent infection via intracellular persistence (Werdan et al., 2014). We conducted a series of experiments to determine if *S. sanguinis* strains possess the ability to adhere to and invade mammalian cells. First, we screened the entire set of *S. sanguinis* mutants for invasion defects on epithelial (HeLa) cells, and further characterized any strain of interest for their adherence properties. In sum, we identified 13 mutants deficient for invasion by plating for CFUs following a gentamicin protection assay (Supplementary Figure S2B). Of these 13 mutants, 7 were also deficient for adherence to HeLa cells (Supplementary Figure S2A). The adherence of each mutant was determined by enumerating the number of bacterial cells per HeLa cell from microscopy images of ca. 100 individual cells.

**FIGURE 4:**
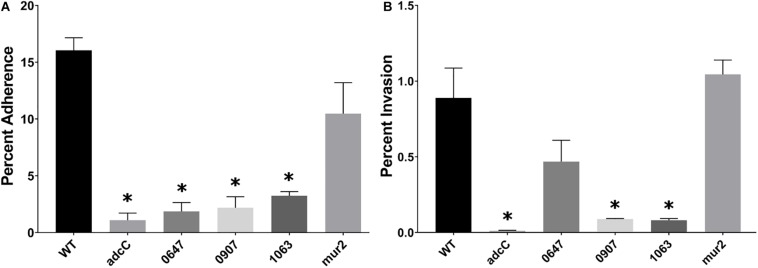
Identification of *S. sanguinis* mutants that display reduced adherence to and invasion of human aortic endothelial cells. **(A)** Adherence of the indicated mutant strains to HAECs. Bacterial cells were incubated with HAECs for 3 h. After removing non-adherent bacteria through washing, cells were fixed with paraformaldehyde and stained with Giemsa. Adherent bacterial cells per HAEC were enumerated via light microscopy and calculated as a percent adherence compared to WT. **(B)** Invasion of HAECs. A representative experiment is shown due to variability observed among assays. After incubation with HAECs for 3 h, cells were washed and incubated for 1 h with medium containing 100 μg/mL gentamicin. HAECs were lifted by a 5-min incubation in trypsin-EDTA and then lysed by addition of Tween 20 in PBS for 20 min at 37°C. Cell lysates were mixed thoroughly and plated for CFU on BHA plates. Statistical analysis was performed using one-way ANOVA and corrected for multiple comparisons against the WT mean using Dunnett’s test. ^∗^*p* < 0.05. Error bars represent the standard error.

Mutant strains compromised for their ability to invade or adhere to HeLa cells were subsequently analyzed for adherence and invasion of immortalized human aortic endothelial cells (HAECs). This cell line has been demonstrated to retain common properties of primary HAECs (Herrera et al., 2017), and is representative of the cell type expected to be encountered by IE-causing bacteria at the site of infection. The ability of each mutant strain to invade or adhere to HAECs was determined as above. We observed four mutant strains that exhibited decreased HAEC adherence (Figure 4A): *adcC*, an ABC zinc transporter involved in biofilm biosynthesis in *S. gordonii*, was reduced greater than 10-fold; *SSA_0647* encodes a hypothetical protein involved in inorganic ion transport, and was reduced ca. eightfold; *SSA_0907* was reduced ca. sixfold and encodes a putative fibronectin-binding protein; and *SSA_1063*, predicted to encode a protein containing peptidoglycan-binding and von Willebrand factor A domains, was reduced ca. fivefold. A trend toward reduced adherence was observed for *mur2*, but this decrease did not reach statistical significance. In the subsequent gentamicin-protection assay, we observed *adcC*, *SSA_0907*, and *SSA_1063* displayed reductions in HAEC invasion of ca. 10-fold (Figure 4B) alongside their reductions in HAEC adherence. In contrast, *SSA_0647* exhibited a trend toward reduced invasion (ca. twofold) without reaching statistical significance, while *mur2* invasion was indistinguishable from WT (Figure 4B). We did not observe any significant changes in chain length among any of the mutants when examined by light microscopy (data not shown).

### Experimental Infective Endocarditis

To determine whether genes required for the *in vitro* phenotypes of tissue culture cell adherence, invasion and platelet binding and aggregation function in the pathogenesis of IE, we selected two mutants for analysis in a New Zealand White rabbit model of left-sided native valve IE. This experimental system models the underlying disease physiology commonly observed in native valve endocarditis (opposed to prosthetic) and left-sided (aortic or mitral). Due to practical concerns including financial costs, we were not able to test every strain of interest. Mutants Δ*SSA_1099::kan* and Δ*mur2*::kan were selected based on predictions from our *in vitro* data and the expected or putative gene function. Mutant *SSA_1099* exhibited marked loss of platelet binding and aggregation properties, suggesting it would lack elements of both adhesion to the injured valve surface and subsequent development of the vegetation. The *mur2* mutant exhibited a defect in the biofilm assay and trended toward decreased adherence on HAECs, suggesting a colonization phenotype distinct from *SSA_1099*. Further, while we did not observe *in vitro* growth defects for any of these mutants, we were uncertain about potential impacts on *in vivo* metabolism involved in transport or efflux (vs. e.g., *adcC*, *SSA_0647, SSA_0874*). We also reasoned it would be more valuable to select genes that have not been investigated in other species (vs. *SSA*_*0907*) and which have a more specific putative function (vs. *SSA_1515*). The development and weight of cardiac vegetations were considered primary disease metrics in our model.

**FIGURE 5:**
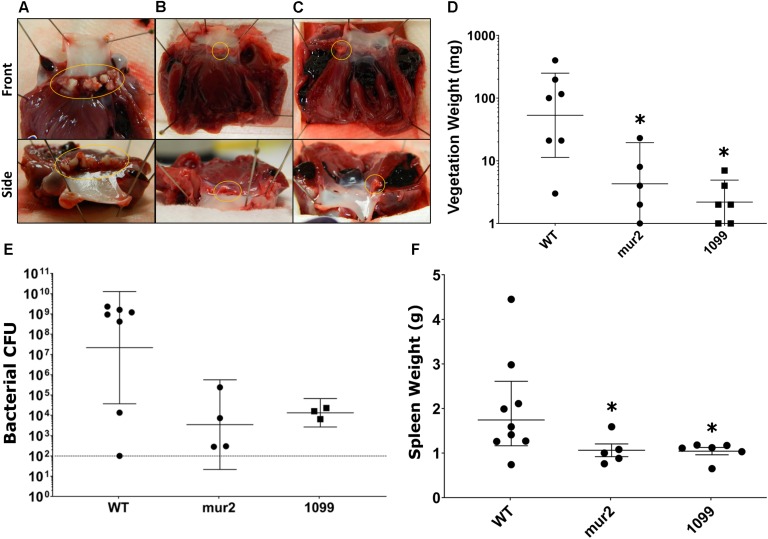
Development of cardiac vegetations and splenomegaly is significantly reduced following infection with *mur2* and *SSA_1099* mutant strains. Representative images of median cardiac vegetations for **(A)** WT, **(B)** *mur2*, and **(C)** *SSA_1099* infected rabbits. Vegetations or thrombi present on the valve are circled in each image. Vegetations observed in WT infected rabbits **(A)** were very large as indicated by average weight, developing over the entire aortic valve (circled), and exhibited a tough, solid composition. In contrast, thrombi or vegetations observed in rabbits infected with either *mur2* or *SSA_1099* mutants **(B,C)** were very small and were usually contained within one aortic cusp (circled). **(D)** Vegetations observed in rabbits infected with mutant strains were determined to produce lesions of significantly reduced weight compared to WT. Lesions from WT-infected rabbits were 15-fold or greater than those observed in rabbits infected with mutant strains. **(E)** CFUs present within the lesion or on the valve surface were reduced compared to WT. The broken line indicates the limit of detection. Due to contamination, CFU data from three *SSA_1099*-infected animals and one from a *mur2*-infected animal could not be determined. **(F)** We also observed a reduction in average spleen size in rabbits infected with the mutant strains, which was significantly different from WT. Statistical analyses were performed using Kruskal-Wallace and corrected for multiple comparisons against the WT mean by controlling the false discovery rate (FDR) via the method of Benjamini, Krieger and Yekutieli. ^∗^, discovery. Error bars represent either the standard error **(F)** or geometric mean with 95% confidence intervals **(D,E)**.

**FIGURE 6:**
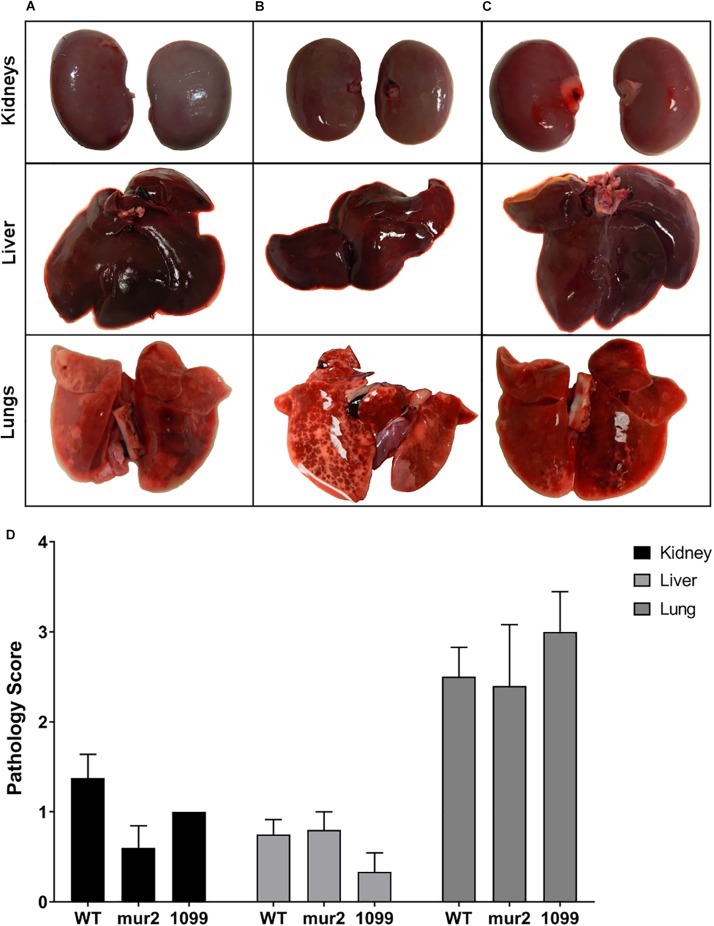
Pathology of distal organs is not significantly different among strains. Gross pathology of the kidney, liver, and lungs from infected rabbits. Representative images of **(A)** WT, **(B)** *mur2*, and **(C)** *SSA_1099*. The pathology scale used for scoring (Supplementary Figure S4) was derived from Kulhankova et al., (unpublished), and organs were scored by three blinded investigators. **(D)** Mutant strains did not exhibit distinct organ pathology compared to WT. Kidneys and liver exhibited moderate pathology on average, characterized by rare, small lesions evident on the surface. By contrast, lungs generally exhibited more severe pathology characterized by multifocal hemorrhaging and necrosis evident on one or both lobes. Statistical analysis was performed using two-way ANOVA against the WT mean and controlling for the FDR. Error bars represent the standard error.

Rabbits infected with WT *S. sanguinis* SK36 were observed to develop cardiac vegetations over the experimental time frame (Figures 5A–D). These large vegetations averaged ca. 122 mg (median: 100 mg) and, with two exceptions, contained >1.0 × 10^8^ CFU viable bacteria per vegetation (Figure 5E). One rabbit presented with only a minor thrombus (3 mg) which could not be determined to contain viable CFUs. Two rabbits succumbed to disease, presumably due to congestive heart failure, prior to euthanasia: one in the afternoon of day six and one in the morning of day seven. Upon necropsy, we observed both rabbits to have developed substantial aortic valve vegetations which would ultimately be the two largest in our experiment (400 and 197 mg). We observed substantial spleen enlargement in about half of the animals infected with WT *S. sanguinis* SK36 (Figure 5F); in total, spleen weights averaged 1.97 g. Splenic inflammation could be predicted (*r*^2^ = 0.64) by vegetation CFU as determined by linear regression (Supplementary Figure S3). However, we could not detect active bacteremia in any animals on days six or seven (data not shown). We also observed moderate pathology in the kidneys and liver and significant pathology in the lungs (Figure 6).

**FIGURE 7:**
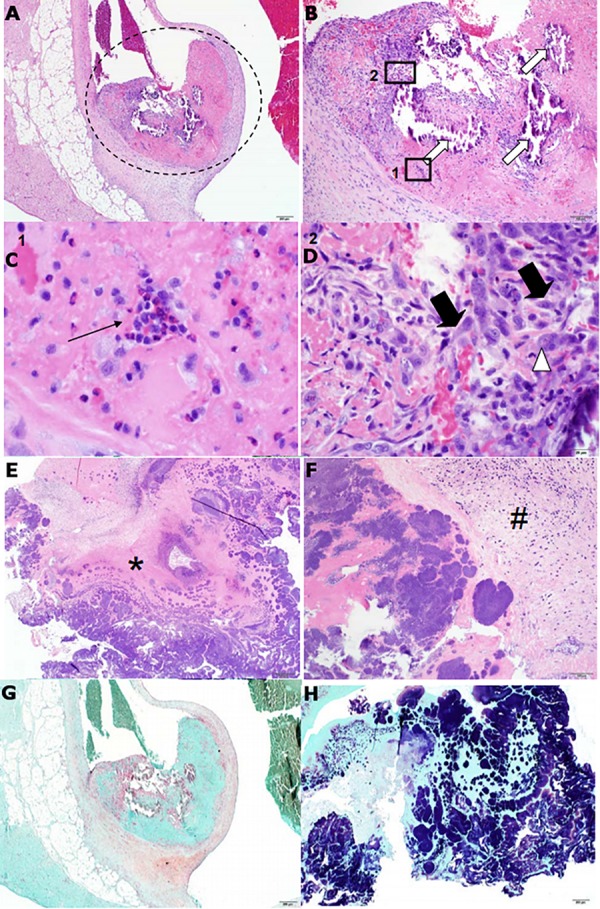
Features of heart valve pathology and cardiac vegetation histopathology from *S. sanguinis*-induced infective endocarditis (IE) in rabbits resemble human pathology. H&E stains of a representative heart valve **(A–D)** and cardiac vegetation **(E–F)** from WT-infected rabbits. **(A)** The presence of a small, focal thrombus (encircled) can be observed originating from the aortic valve. **(B)** Enhanced view of the encircled. The thrombus is composed predominantly of eosinophilic (pink) fibrin and contains multifocal areas of basophilic (blue) mineralization (white arrows). Also apparent are multifocal pockets of heterophils (Box 1) and loosely organized fibroblasts accompanied by macrophages (Box 2). **(C)** Enhanced view of Box 1. Focus of heterophils (thin black arrow), the primary phagocytic cell in rabbits. **(D)** Enhanced view of Box 2. Fibroblasts (thick black arrows) and large, multinucleated macrophage (white arrowhead). **(E)** Tissue of the cardiac vegetation is composed of a network of eosinophilic fibrin (asterisk) intermixed with necrotic cellular debris and abundant, basophilic colonies of coccoid bacteria. **(F)** Presence of a thin band of plump fibroblasts (hash) along with dispersed, variable inflammatory cells. **(G)** Gram stain of the valve could not detect the presence of bacteria, while **(H)** gram stain of the vegetation revealed large quantities of gram positive, coccoid bacteria.

Histopathological analysis of two rabbit hearts from an independent experiment and an isolated vegetation segment from a previous experiment was performed by a veterinary pathologist. Heart sections were characterized by a focal thrombus originating at the aortic valve; thrombi were comprised of fibrin, multifocal mineralization, and loosely organized, multifocal pockets of fibroblasts and inflammatory cells (Figures 7A–D,G). Overall, these observations are indicative of subacute, and approaching chronic, thrombosis. The vegetative mass was composed predominantly of fibrin and intermixed with dense, occasionally large, gram positive bacterial colonies (Figures 7E–F,G).

In contrast to our WT observations, infection with Δ*SSA_1099::kan* and Δ*mur2::kan* produced only small vegetations and thrombi (Figures 5A–D). Homogenates of these lesions, or valve scrapings from those animals without apparent cardiac vegetation, typically yielded less than 1 × 10^5^ CFUs (Figure 5E). All rabbits in these infection groups survived to the end of the experiment. Spleen size in these animals was reduced (Figure 5F), while pathology of the liver, kidney, and lungs was indistinguishable (Figure 6) compared to WT.

## Discussion

Oral streptococci are significant etiological agents of IE worldwide, particularly in developing and resource-limited nations where rheumatic heart disease is a prevailing predisposing condition. Despite their historical association with the disease, virulence factors of oral streptococci contributing to the pathogenic process remain under-characterized and many putative virulence genes have not been investigated experimentally. We believe investigations directed toward addressing these limitations will ultimately engender improved clinical outcomes, especially in regions where diagnostic and treatment options are limited. For instance, vaccines to prevent infection would preempt complications from late diagnosis or inaccurate antimicrobial therapy and identify novel therapeutic targets to complement the standard antibiotic regimen (Thuny et al., 2012; Cahill et al., 2017). The goal of our present research is to identify virulence factors mediating pathogenesis of *S. sanguinis*, one of the most common oral *Streptococcus* species causing IE. We expect these investigations to facilitate further research toward the development of improved therapeutics and inform clinical management of IE.

We constructed an initial pool of 128 unique mutants containing knockouts for genes predicted to encode virulence factors. These genes (Supplementary Table S1) were selected through a combination of literature surveys and computational annotation of the *S. sanguinis* SK36 genome available on NCBI. Most putative virulence factors identified are predicted to function in adhesion/aggregation to host cells and cell wall metabolism. Genes predicted to function in biofilm formation, lipid, sugar, nucleic acid, and metal metabolism, redox processes, immune evasion, quorum sensing, and transcriptional regulation were also identified. Interestingly, several genes were annotated as putative toxins or associated with their function, which is generally considered absent in oral streptococci. A list of genes identified in our *in vitro* investigations and their known or putative function is presented in Table 1.

**TABLE 1:** Summary of *S. sanguinis* genes identified using *in vitro* assays.

**Gene designation**	**Known or putative function**	**Biofilm**	**Platelet Adh**	**Platelet Agg**	**HAEC Adh**	**HAEC Inv**
*pcsB*	Peptidoglycan hydrolase	−	+	+	−	−
*adcC*	ABC transporter, Zn porter	−	−	−	+	+
*SSA_0647*	Inorganic ion transport	−	−	−	+	−
*srpA*	Platelet-binding glycoprotein	−	+	+	−	−
*SSA_0837*	Glycosyltransferase	−	+	+	−	−
*SSA_0841*	Accessory secretory protein	−	+	−	−	−
*murN*	Peptidoglycan cross-linking	−	+	±	−	−
*SSA_0874*	Metabolite efflux	+	−	+	−	−
*SSA_0907*	Fibronectin-binding protein A	−	−	+	+	+
*SSA_1063*	Peptidoglycan-binding, vWFA-domain protein	−	−	−	+	+
*mur2*	Peptidoglycan hydrolase	+	−	−	±	−
*SSA_1099*	RTX-like adhesin	±	+	+	−	−
*SSA_1101*	RTX export component	−	+	−	−	−
*SSA_1129*	Iron transport lipoprotein	−	+	+	−	−
*srtA*	Sortase A	−	+	+	−	−
*SSA_1515*	Cell wall polysaccharide	±	−	−	−	−
*hlyX*	CBS domain adhesin	−	+	+	−	−
*nrdD*	Anaerobic ribonucleoside-triphosphate reductase	−	+	+	−	−

*Genes with novel associations to virulence-related phenotypes are colored light blue. +, phenotype; −, no phenotype; ±, intermediate phenotype.*

We identified four genes whose deletion resulted in decreased biofilm formation *in vitro* (Figure 1). Biofilm formation in this assay is expected to involve at least two bacterial factors: adherence to the microtiter surface and production of EPS. It is also possible that moderate adherence defects could be masked by massive *in vitro* EPS production. We therefore considered biofilm formation in this assay to be primarily indicative of EPS production and secretion. We observed that strains with the two largest reductions in biomass formation were deleted in either *SSA_1515* or *mur2*. *SSA_1515* is part of a locus encoding homologs to cell wall polysaccharides in other oral streptococci which are known to mediate intra-bacterial aggregation (Xu et al., 2007). Thus, *SSA_1515* likely contributes to biofilm formation by promoting cell-cell adhesion between organisms or by serving as a trigger for EPS production. *mur2*, encoding a putative PG hydrolase, may influence biofilm formation through multiple mechanisms. PG hydrolases are known modulators of biofilm development in other gram-positive species where they may function to expose adhesins and mediate adherence, cell morphology, and autolytic processes for the release of extracellular DNA. Strains with mutations in two additional genes, *SSA_0874* and *SSA_1099*, exhibited more modest biofilm defects in our assay. A predicted sulfate export transporter is encoded by *SSA_0874* and potentially contributes to metabolism during biofilm growth. *SSA_1099* encodes a predicted RTX-like protein of 1,477 amino acids. Repeat-in-toxin (RTX) proteins may function as cytolysins or adhesins (Linhartová et al., 2010) and have been exclusively characterized in gram-negative organisms. Considering *S. sanguinis* has not been observed to possess any hemolytic or cytolytic activities, we conclude that this protein functions as an adhesin in *S. sanguinis*, as our data strongly suggests. Given the large size of this protein, it is likely *SSA_1099* contributes to biofilm architecture by mediating cell-to-cell interactions or multiple binding activities as has been observed for other RTX adhesins (Satchell, 2011; Dolev et al., 2016; Guo et al., 2019).

Mutants deficient in binding to platelets are expected to possess reduced potential for adherence to damaged endocardium, while reduced aggregation would be expected to reduce vegetation growth and development. Consistent with these expectations, mutants in *srtA* or *srpA* – which we identify here – have been shown to be attenuated in animal models of IE (Xiong et al., 2008; Turner et al., 2009). Nine mutants from our library were determined to exhibit deficiencies in platelet binding (Figure 2), and eight of these were concomitantly reduced in platelet aggregation (Figure 3). Since prior binding to platelets is often mechanistically associated with the ability to subsequently aggregate platelets, we cannot conclude the dual phenotypes observed here are independent activities without further investigation. Two of these genes are predicted to encode proteins functioning in cell envelope metabolism, including *pcsB* (PG hydrolase) and *murN* (PG cross-linking), suggesting they function to modulate platelet interaction via adhesin accessibility and cell wall architecture. *murN* was unique among our platelet interactivity mutants in that it exhibited an intermediate phenotype in our platelet aggregation assay. This discrepancy suggests platelet binding and aggregation phenotypes mediated by *murN* are at least partially decoupled. Predicted glycosyltransferase *SSA_0837* may provide essential modifications to platelet-interacting proteins. The *SSA_0841* strain is the only binding mutant that exhibited an opposite phenotype for platelet aggregation. *SSA_0841* in *S. sanguinis* possesses significant similarity to the Asp5 accessory protein in *S. parasanguinis* and *S. gordonii* where it functions in exporting the SrpA protein. Previous work in *S. gordonii* has indicated Asp5 shares functionality with another protein, where the absence of Asp5 results in only partial loss of SrpA surface expression (Takamatsu et al., 2005; Bandara et al., 2016). In our results with *S. sanguinis*, the *srpA* mutant is deficient for both platelet binding and aggregation in our assays (Figures 2, 3) while *SSA_0841* is defective in platelet binding but displays wild type (WT) levels of aggregation. These results suggest the interactions mediated by surface SrpA and necessary for functional adhesion to and aggregation of human platelets are quantitatively distinct.

Iron transport mediated by the lipoprotein predicted by *SSA_1129* may serve to provide adequate iron levels for use as cofactors in platelet-interacting proteins. Surprisingly, the anaerobic ribonucleotide reductase, *nrdD*, was also identified in these assays. *nrdD* is required for growth under anaerobic conditions where it reduces ribonucleotides to deoxyribonucleotides and is essential for virulence (Paik et al., 2005). While we did not observe any significant growth deficiencies under our assay conditions, it is possible subtle growth differences from the absence of the *nrdD* gene product have an indirect effect on proteins that interact with platelets. The largest decrease in platelet binding was observed for *hlyX*, which the annotation indicates encodes a hemolysin-like protein. Considering the assay that detected this mutant uses immobilized bacteria as a substrate for human platelets, it is unlikely this protein is predominantly secreted. Most likely, since no hemolytic activity is observed for *S. sanguinis* or oral streptococci in general, we believe this protein functions as an adhesin. *SSA_1099* and its putative complex protein, *SSA_1101*, both exhibit substantial reductions in platelet binding and aggregation capability. Like *hlyX*, the results here suggest *SSA_1099* is at least partly localized to the cell surface. Additionally, *SSA_1101*, predicted to encode a membrane fusion protein involved in the transport of *SSA_1099*, is also unable to bind to and aggregate platelets, indicating that the activity of this protein is likely necessary for proper localization of *SSA_1099*. Based on the function described here for *SSA_1099* and *SSA_1101*, and the lack of hemolytic activity associated with oral streptococci, we propose naming these genes, and the intervening *SSA_*1100, platelet-binding RTX, or *pbrABC*.

A preliminary screen for deficiencies in either adherence or invasion on HeLa cells was performed which identified 13 mutants for further investigation (Supplementary Figure S2). We subsequently identified four mutants compromised in adherence to HAECs (Figure 4A), three of which were also reduced for invasion (Figure 4B). *adcC* encodes a putative zinc transporter as part of an operon orthologous to one identified in *S. gordonii.* Components of this operon have been shown to modulate biofilm formation in both *S. gordonii* and *S. sanguinis* (Loo et al., 2003; Ge et al., 2008), suggesting zinc availability influences adhesion to host cells. A hypothetical protein predicted to be involved in inorganic ion transport is encoded by *SSA_0647*. While this mutant is severely compromised in adhesion, its ability to invade is only partially inhibited (compare Figures 4A,B), suggesting this protein is disproportionately involved in cell adherence. *SSA_0907* encodes a putative fibronectin-binding protein present in other oral streptococci, likely mediating adhesion through direct interaction with fibronectin molecules produced by HAECs. A predicted cell wall-binding protein containing a von Willebrand factor (vWF) type A domain encoded by *SSA_1063* was identified as reduced for both adhesion and invasion. Considering vWF is a common marker of endothelial cells, it is likely this protein directly binds vWF to mediate adhesion. The putative PG hydrolase *mur2* could not be determined to have an adhesion phenotype distinct from WT, though it exhibited a trend toward reduced adhesion. In contrast, our invasion results indicate *mur2* has no discernable deviation from WT.

Infective endocarditis caused by viridans streptococci is a chronic, subacute disease commonly localized to the aortic or mitral valve and capable of development on both native and prosthetic surfaces. While the epidemiology of streptococcal IE tends to favor the native valve condition (Hill et al., 2007; Murdoch et al., 2009), most experimental systems (rabbit or rodent) have induced aortic injury via persistent (long-term) catheterization of the valve (Durack et al., 1973; Munro and Macrina, 1993; Ge et al., 2008; Das et al., 2009; Callahan et al., 2011; Fan et al., 2012; Crump et al., 2014). These models introduce a significant long-term stress to heart function as well as an abiotic surface to the cardiac environment, both of which may lead to results that vary from conditions of a native valve surface (Schlievert et al., 1998). While these published investigations are of considerable importance and data acquired using these models has provided valuable insights toward understanding mechanisms of IE caused by oral streptococci, we view these models as distinct from the native valve condition. Indeed, predisposing conditions, sequelae, and epidemiology observed in prosthetic valve IE are often distinct from native valve IE (Romano et al., 2004; Fowler et al., 2005; Murdoch et al., 2009).

Rabbits infected with *S. sanguinis* SK36 reliably developed cardiac vegetations of considerable size compared to our mutant strains (Figures 5A–D). Only one rabbit infected with a mutant strain, *mur2*, developed a vegetation >20 mg and contained greater than average CFU. While it was possible to quantify smaller lesions present in mutant-infected rabbits, their diminutive size and low CFUs (Figures 5D,E) made it difficult to identify with confidence whether these lesions were bacteria-induced vegetative growths or simply colonized thrombi developed from mechanical injury. Considering bacterial colonization could be demonstrated in these conditions, we conceive of vegetation sizes <10 mg as primarily indicative of thrombosis at the site of aortic injury. Using this hypothesis, our results suggest both the *SSA_1099* and *mur2* mutants are almost completely compromised in their ability to cause IE. However, since one rabbit infected with the *mur2* mutant was observed to develop a small vegetation, this mutant may be capable of causing IE under certain conditions. Based on these data, we hypothesize SSA_1099 contributes to pathogenesis via binding to matrix proteins and/or platelets at the site of valvular injury and via growth of the vegetative lesion, while *mur2* may promote colonization and resistance to immune clearance by mediating efficient endothelial contact and development of stable “biofilm” architecture.

While we could reliably expect to find vegetations in rabbits infected with WT, we did observe a range a vegetation sizes observed in WT-infected rabbits. This is likely due to the outbred nature of our rabbits or inconsistencies in inducing aortic lesions. Over the course of our experiments, one rabbit infected with *S. sanguinis* SK36 WT developed only a minor aortic lesion and possessed no detectable CFU on the valve surface. In contrast, we were able to recover CFUs from both of our mutant strains even when vegetations were not present (Figures 5D,E), suggesting bacteremia was not successfully initiated following surgery in this rabbit.

Histopathology analysis provides evidence for the formation of a fibrin-composed thrombus on the aortic valve following catheter-induced injury (Figure 7), which is consistent with the predisposing endothelial damage observed in humans (Holland et al., 2016). The presence of mineralization has also been associated with chronic valve infection in humans (Thiene and Basso, 2006) and with streptococcal IE in pigs (Jensen et al., 2010). The vegetative mass itself was composed of a fibrin meshwork heavily embedded with colonies of *S. sanguinis* (Figure 7G), further consistent with observations of cyclical platelet-fibrin deposition and bacterial growth during development of the vegetation (Holland et al., 2016). Interestingly, we observed the vegetations in WT-infected rabbits to exhibit marked toughness, which is consistent with thrombi having a heavy fibrin composition. In contrast, and in our experience, vegetations caused by *S. aureus* are generally softer and more malleable. Together, these data suggest that the rabbit model used in our infection studies is representative of native valve human IE caused by viridans group streptococci.

## Conclusion

We have identified several genes with important phenotypes in virulence-associated *in vitro* assays. Two of these genes, *SSA_1099* and *mur2*, were determined to also possess substantially reduced virulence in an animal model of native valve endocarditis. Further, we have demonstrated the efficacy of the aortic injury procedure as a model for studying the role *S. sanguinis* virulence factors in endocarditis.

## Data Availability Statement

All datasets generated for this study are included in the article/Supplementary  Material.

## Ethics Statement

The studies involving human participants were reviewed and approved by human platelets were acquired from the University of Iowa DeGowin Blood Center as platelet-rich plasma (PRP). We have received approval from the University of Iowa Institutional Review Board to use human blood components under protocol #201902770. All samples used for this work were anonymized. The patients/participants provided their written informed consent to participate in this study. The animal study was reviewed and approved by all animal experiments were performed in accordance with the protocols of the Institutional Animal Care and Use Committee of the University of Iowa (protocol #1106138). The University of Iowa is a registered research facility with the U.S. Department of Agriculture (USDA) and complies with the Animal Welfare Act and Regulations (AWA/AWR). The facility holds an Animal Welfare Assurance through the Office of Laboratory Animal Welfare (OLAW) and complies with PHS Policy. Additionally, the facility is accredited through the Association for Assessment and Accreditation of Laboratory Animal Care International (AAALACi) and is committed to comply with the Guide for the Care and Use of Laboratory Animals.

## Author Contributions

AM, AR, PT, and BJ conceived and designed the experiments. AM, BM, AR, PT, and MS performed the experiments. KK and WS-P performed and directed the rabbit surgeries. AF assisted with the rabbit surgeries and supervised the animal monitoring during anesthesia. AM, BM, and AR analyzed the data. AM and BJ wrote the manuscript.

## Conflict of Interest

The authors declare that the research was conducted in the absence of any commercial or financial relationships that could be construed as a potential conflict of interest.
