# Supplementary material for: Association of Novel Streptococcus sanguinis Virulence Factors With Pathogenesis in a Native Valve Infective Endocarditis Model
Source: Front Microbiol. 2020 Jan 31;11:10. doi: 10.3389/fmicb.2020.00010 (PMC7005726; doi:10.3389/fmicb.2020.00010)
Supplement: Supplementary file 1 [file Data_Sheet_1.docx]

**
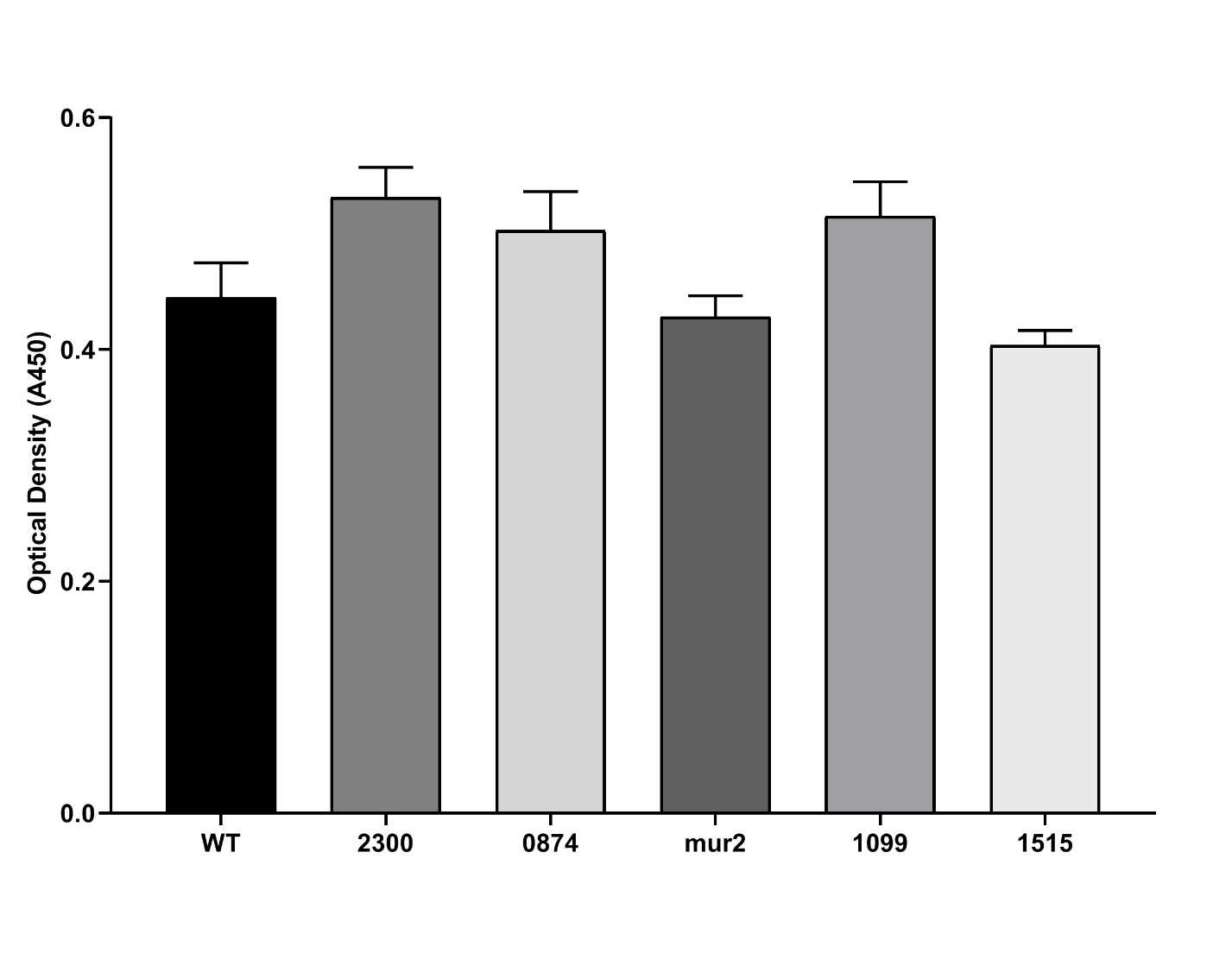
SFig. 1. Cell growth of *S. sanguinis* strains in BM after 24 hours.** To ensure that any differences in biofilm formation were not attributable to differences to growth in the biofilm media, we grew *S. sanguinis* strains statically in the anaerobic chamber and measured the optical density after 24 hours. Strains that were not statistically different from wild type were compared in biofilm experiments. Assays were performed in triplicate and three experiments were performed. Statistical analysis was performed using one-way ANOVA. * = p < 0.05. Error bars represent the standard error.

**
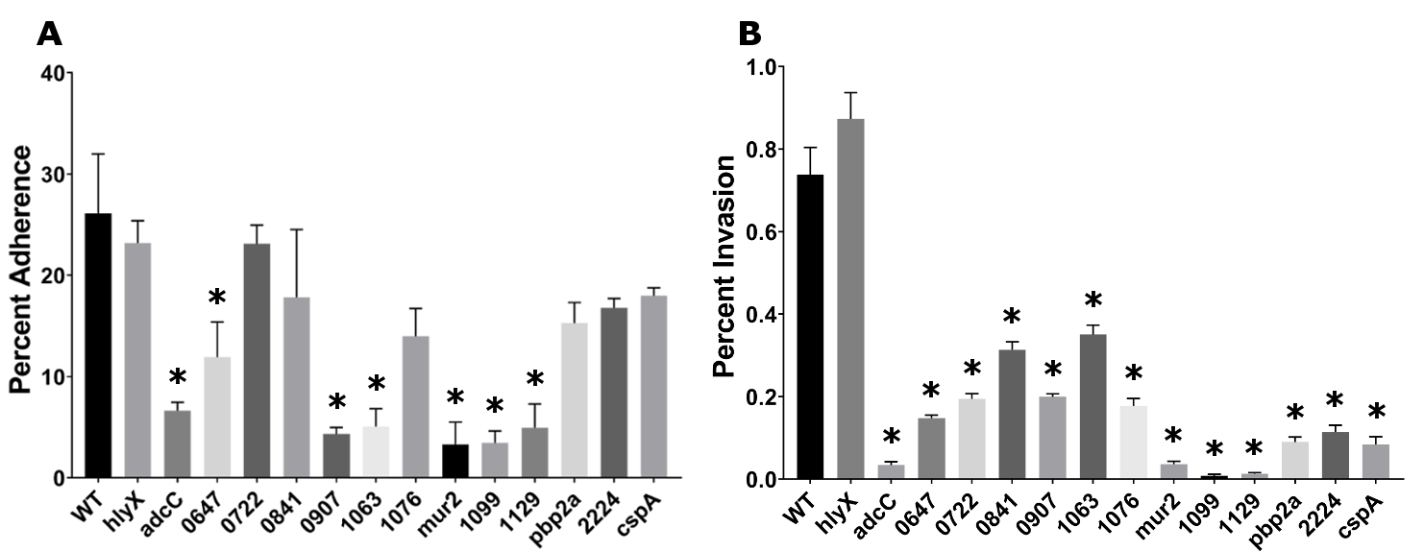
SFig 2. Putative virulence genes associated with (A) adherence and (B) invasion of HeLa cells.** As a preliminary screen to identify *S. sanguinis* mutants that had reduced ability to adhere to and invade human cells, all 128 mutants were examined in tissue culture assays. Due to variability among assays, a representative experiment is shown. Mutants with reduced HeLa cell adherence and HeLa cell invasion are shown in panels A and B, respectively. All other mutants displayed WT levels of adherence and invasion or had significantly reduce growth rates that precluded their evaluation in these assays. Assays were performed in triplicate and three experiments were performed. Statistical analysis was performed using one-way ANOVA. * = p < 0.05. Error bars represent the standard error.


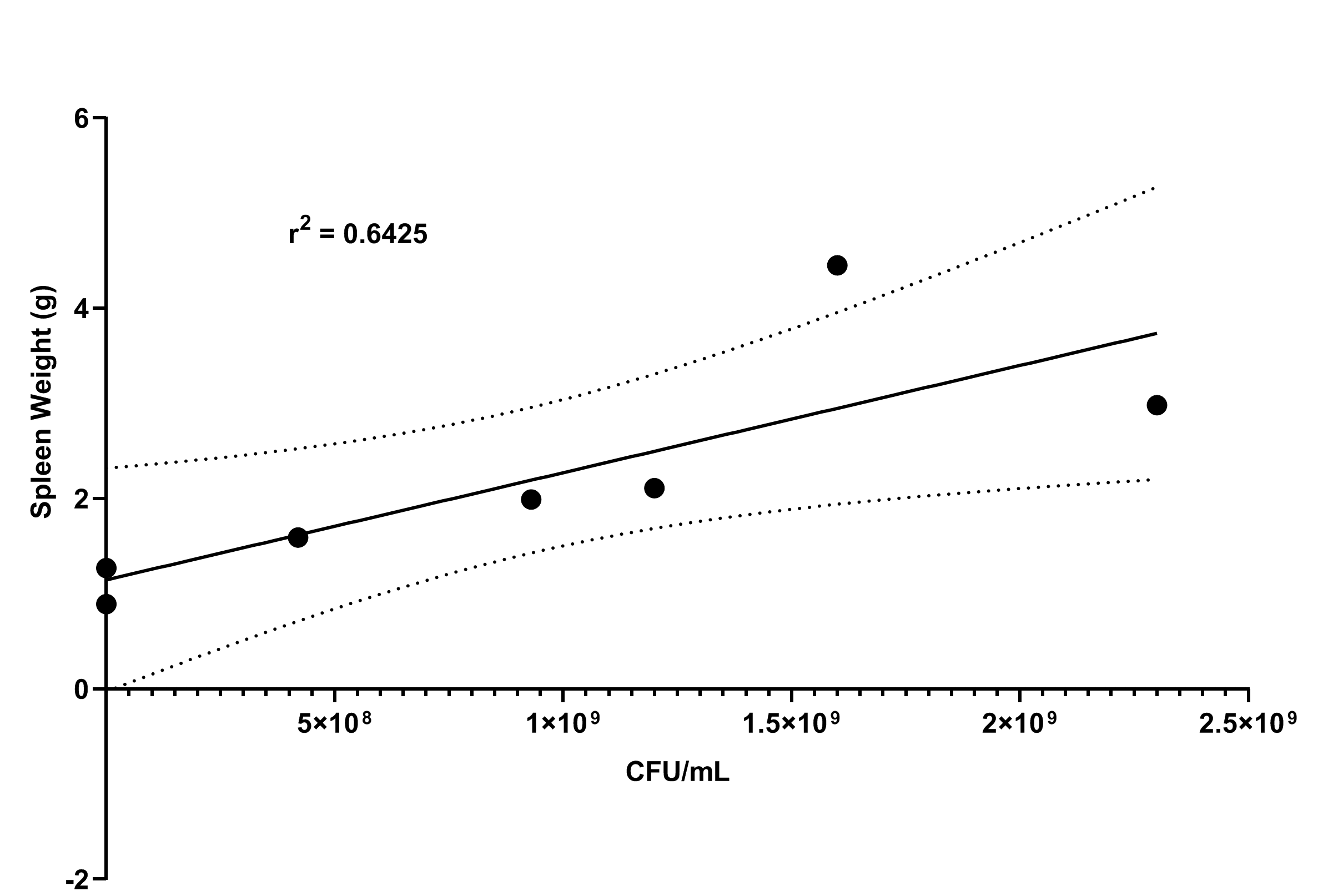
**SFig 3. Linear regression analysis of WT vegetation or valve CFU and spleen size.** Data shows the correlation between the number of wild-type bacteria recovered from lesions or vegetations on the aortic valve and the corresponding size of the spleen isolated from the same rabbit. The broken lines above and below the solid line are the 95% confidence intervals for the data set.


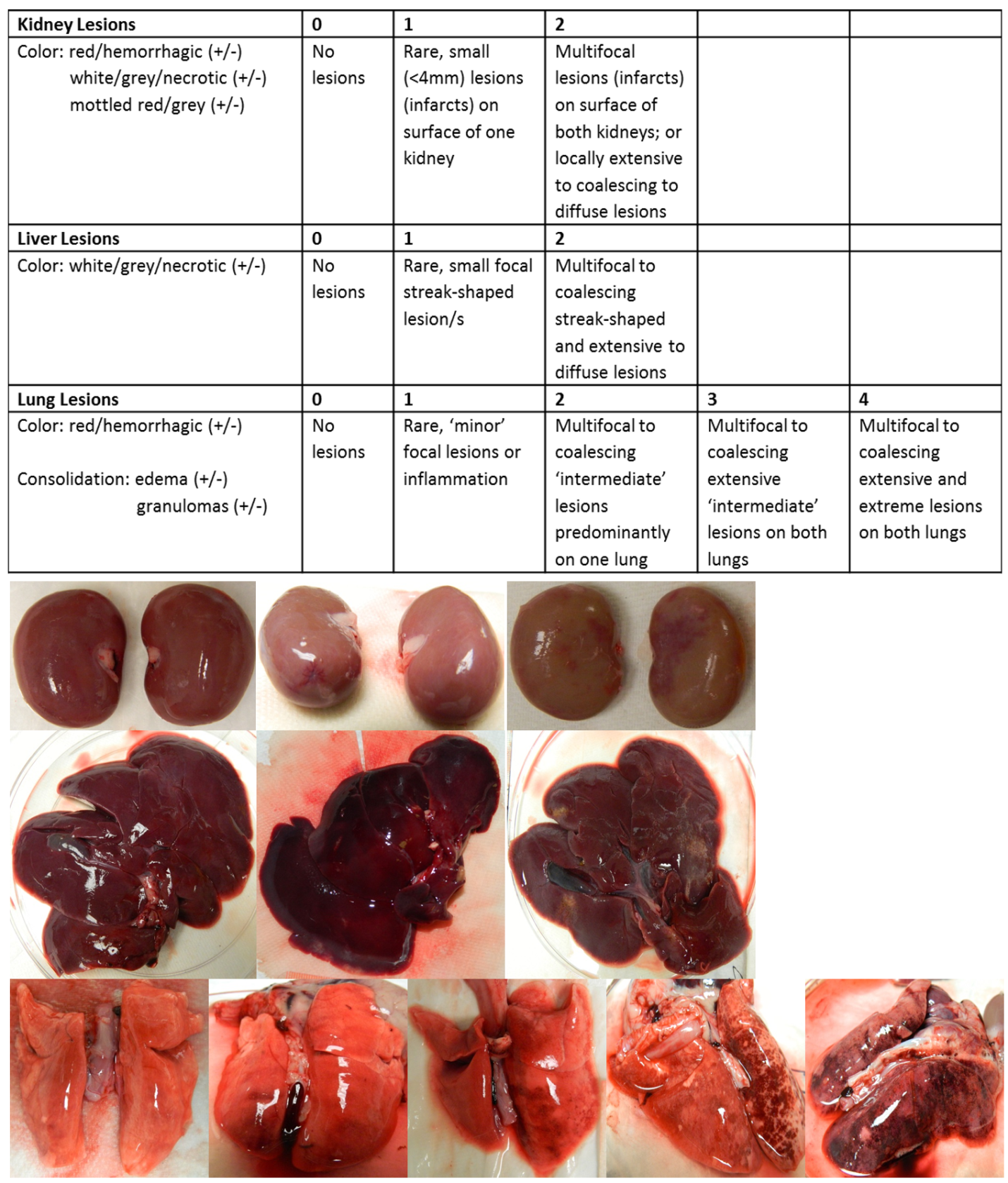
**SFig 4. Pathology scale used to score peripheral organs from rabbits infected with *S. sanguinis*.** The table portion of the figure shows the criteria for kidney, liver and lungs pathology scoring effects *S. sanguinis* infection. Below the table are representative organs that received the score corresponding to the column in table above - far left organs would have no lesions with organs displaying progressively more pathology moving from left to right. Adapted from Kulhankova K, Salgado-Pabón W et al., manuscript in preparation.

**STable 1**. **Table of 128 putative virulence genes.**

| **Locus tag** | **Product** | **Property** | **Gene Name** | **Reference** |
| --- | --- | --- | --- | --- |
| **SSA_0019** | secreted antigen GbpB/SagA; peptidoglycan hydrolase; PcsB protein precursor | Binding to glucans (ortholog in *S. mutans*) | pcsB | (Moraes et al., 2014; Xu et al., 2007) |
| **SSA_0022** | surface protein cell wall anchor | Sortase; CWA processing | srtB | (Xu et al. 2007) |
| **SSA_0030** | phosphoribosylformylglycinamidine synthase | Purine synthesis, biofilm reduction |  | (Ge et al., 2008) |
| **SSA_0046** | adenylosuccinate lyase | Purine synthesis, biofilm reduction | purB | (Ge et al., 2008; Paik et al., 2005; Xu et al., 2007) |
| **SSA_0135** | multiple antibiotic resistance operon transcriptional repressor (MarR) | adc operon for biofilm synthesis (*S. gordonii*) | adcR | (Xu et al. 2007) |
| **SSA_0136** | ABC transporter, Zn porter | adc operon for biofilm synthesis (*S. gordonii*) | adcC | (Xu et al. 2007) |
| **SSA_0137** | ABC transporter (permease), Zn porter | adc operon for biofilm synthesis (*S. gordonii*) | adcB | (Xu et al. 2007) |
| **SSA_0138** | metal-binding (Zn) permease | adc operon for biofilm syntheis, biofilm reduction | adcA | (Ge et al., 2008; Turner et al., 2009) |
| **SSA_0192** | acetate kinase |  | ackA | (Chen et al., 2012; Moraes et al., 2014) |
| **SSA_0227** | collagen-binding surface protein | Collagen binding domain |  | (Xu et al. 2007) |
| **SSA_0236** | recombination factor protein RarA | cell wall associated adhesion | *CshA* | (Black et al., 2004) |
| **SSA_0260** | manganese/Zinc ABC transporter substrate-binding protein | Virulence for endocarditis | ssaB | (Das et al., 2009; Herzberg, 1996; Turner et al., 2009b; Xu et al., 2007) |
| **SSA_0261** | ABC-type Mn2+/Zn2+ transport systems, permease component | similar to fim operon in parasanguinis…crucial for survival in macrophages | ssaC | Computational Prediction |
| **SSA_0262** | ABC-type Mn/Zn transporter, ATP-ase component | similar to fim operon in parasanguinis…crucial for survival in macrophages | ssaA | Computational Prediction |
| **SSA_0303** | surface protein C | Binding to collagen, dentinal tubule invasion, coag with P. gingivalis, binding to salivary agglutinin (ortholog in *S. gordonii*); cariogenicity, binding to fibronectin, fibrinogen, virulence for bacteremia (ortholog in *S. mutans*); homology to glucan binding in *S. mutans* | sspC | (Xu et al. 2007, Moraes et al. 2014) |
| **SSA_0331** | C3-degrading proteinase | C3 degradation | cppA | (Xu et al. 2007) |
| **SSA_0332** | Hypothetical Protein | defense mechanisms |  | Computational Prediction |
| **SSA_0374** | peptide methionine sulfoxide reductase msrA/msrB | platelet binding (ortholog in *S. gordonii*) |  | (Herzberg et al., 2005; Xu et al., 2007) |
| **SSA_0391** | pyruvate oxidase |  | spxB | (Chen et al., 2012; Ge et al., 2016; Moraes et al., 2014) |
| **SSA_0453** | Type II secretory pathway, pullulanase PulA glycosidase | similar to adherence protein in iriae |  | Computational Prediction |
| **SSA_0574** | Hypothetical Protein | possible membrane associated transporter |  | Computational Prediction |
| **SSA_0629** | Hypothetical Protein | defense mechanisms |  | Computational Prediction |
| **SSA_0647** | Hypothetical Protein | inorganic ion transport |  | Computational Prediction |
| **SSA_0722** | Hypothetical Protein | possible membrane-associated peptidase |  | Computational Prediction |
| **SSA_0805** | collagen-binding surface protein | Collagen binding |  | (Xu et al. 2007) |
| **SSA_0819** | Hypothetical Protein | cell envelope biogenesis |  | Computational Prediction |
| **SSA_0829** | platelet-binding glycoprotein | Platelet binding; HUVEC cell invasion, endocarditis virulence, binding to salivary agglutinin (ortholog in *S. gordonii*); adhesion to saliva-coated hydroxyapatite (ortholog in *S. parasanguinis*) | srpA | (Xu et al. 2007) |
| **SSA_0830** | glycosyltransferase | homology to glycosylation and export of 0829 in *S. parasanguinis* and *S. gordonii* |  | (Xu et al. 2007) |
| **SSA_0831** | Hypothetical Protein | cell envelope biogenesis, homology for glycosylation and export of 0829 in *S. parasanguinis* and *S. gordonii* |  | (Xu et al. 2007) |
| **SSA_0837** | glucosyltransferase | Homology for glycosylation and export of 0829 in *S. parasanguinis* and *S. gordonii* |  | (Xu et al. 2007) |
| **SSA_0841** | hypothetical protein | homology to glycosylation and export of 0829 |  | (Xu et al. 2007) |
| **SSA_0861** | Hypothetical Protein | defense/possible methicillin resistance | murN | Computational Prediction |
| **SSA_0874** | Hypothetical Protein | possible metabolite efflux protein |  | Computational Prediction |
| **SSA_0905** | CshA-like fibrillar surface protein B | adherence to other bacterium and fibronectin, HUVEC cell invasion, adherence to platelets (ortholog in *S. gordonii*) | crpB | (Xu et al. 2007, Herzberg 1996) |
| **SSA_0906** | CshA-like fibrillar surface protein C | adherence to other bacterium and fibronectin, HUVEC cell invasion, adherence to platelets (ortholog in *S. gordonii*) | crpC | (Xu et al. 2007, Herzberg 1996) |
| **SSA_0907** | fibronectin-binding protein A | adherence to fibronectin (orthologs in *S. gordonii* and *S. pneumoniae*) |  | (Xu et al. 2007) |
| **SSA_0937** | transcriptional regulator Spx |  | spxA | (Chen et al. 2012) |
| **SSA_0955** |  | ~60% identity to M1 protein for fibrinogen binding |  | Computational Prediction |
| **SSA_0956** | surface protein D | Binding to collagen, dentinal tubule invasion, coag with P. gingivalis (ortholog in *S. gordonii*); homology to glucan binding in *S. mutans* | sspD | (Xu et al. 2007, Moraes et al. 2014) |
| **SSA_1006** | dextransucrase | synthesis of dextrans from glucose, allows adherence to fibrin-platelet aggregates | gtfA | (Xu et al. 2007) |
| **SSA_1019** | collagen-binding surface protein | Collagen binding |  | (Xu et al. 2007) |
| **SSA_1023** | von Willebrand factor-binding protein precursor | Von Willebrand binding (ortholog in *S. lugdunensis*) |  | (Xu et al. 2007) |
| **SSA_1044** | homoserine kinase | endocarditis virulence, biofilm reduction | thrB | (Ge et al. 2008, Paik et al. 2005, Xu et al. 2007) |
| **SSA_1063** | peptidoglycan binding domain-containing protein | Von Willebrand factor type A domain, cd00198 |  | (Xu et al. 2007) |
| **SSA_1069** | lipoprotein signal peptidase | LP processing | lspA | (Turner et al., 2009a; Xu et al., 2007) |
| **SSA_1076** | Hypothetical Protein | Lipid metabolism? |  | Computational Prediction |
| **SSA_1080** | fructose operon transcriptional repressor | inducible fructose phosphotransferase operon related to biofilm formation (ortholog in *S. gordonii*) | fruR | (Xu et al. 2007) |
| **SSA_1081** | 1-phosphofructokinase | inducible fructose phosphotransferase operon related to biofilm formation (ortholog in *S. gordonii*) | fruB | (Xu et al. 2007) |
| **SSA_1082** | PTS system, fructose specific II ABC components | inducible fructose phosphotransferase operon related to biofilm formation (ortholog in *S. gordonii*) | fruA | (Xu et al. 2007) |
| **SSA_1095** | peptidoglycan hydrolase | similar to autolysin in iriae | mur2 | Computational Prediction |
| **SSA_1099** | calcium binding hemolysin-like protein | RTX toxin (ortholog in *P. syringae*) |  | (Xu et al. 2007) |
| **SSA_1100** | hemolysin exporter, ATPase component | component of RTX transport (similar to plant-pathogenic pseudomonads) |  | (Xu et al. 2007) |
| **SSA_1101** | multidrug resistance efflux pump/hemolysin secretion transmembrane protein | component of RTX transport (similar to plant-pathogenic pseudomonads) |  | (Xu et al. 2007) |
| **SSA_1106** | IgA-specific metalloendopeptidase | igA, sortase A, colonization, virulence | iga | (Black et al., 2004; Xu et al., 2007) |
| **SSA_1112** | cell wall surface anchor family protein | similar to Pneumococcal adhesin that interacts with Human Thrombospondin-1 | (pavB in pneu) | Computational Prediction |
| **SSA_1129** | periplasmic iron transport lipoprotein | association with TAT |  | (Xu et al. 2007, Turner et al. 2009) |
| **SSA_1130** | Iron-dependent peroxidase | association with TAT; encodes both concensus and signal motifs |  | (Xu et al. 2007) |
| **SSA_1131** | high-affinity Fe 2+/Pb2+ permease | association with TAT |  | (Xu et al. 2007) |
| **SSA_1132** | TatC, sec-independent protein translocase | Homolog (*S. thermophilus)* of TAT | tatC | (Xu et al. 2007) |
| **SSA_1133** | TatA, sec-independent protein secretion pathway component | Homolog (*S. thermophilus)* of TAT | tatA | (Xu et al. 2007) |
| **SSA_1157** | PvaA-like protein | similar to autolysin in iriae |  | Computational Prediction |
| **SSA_1219** | sortase | Sortase A, oral adhesion (ortholog in *S pneumoniae* and *S. gordonii*); cariogenicity (ortholog in *S. mutans*); CWA processing | srtA | (Xu et al. 2007) |
| **SSA_1234** | 5'-nucleotidase | reduction in mean CFUs, reduction in vegetations |  | (Fan et al., 2012) |
| **SSA_1240** | orotate phosphoribosyltransferase | pyrimidine synthesis, biofilm reduction | pyrE | (Ge et al. 2008) |
| **SSA_1324** | ceramide glucosyltransferase |  |  | (Moraes et al. 2014) |
| **SSA_1340** | Zn/Mn ABC-type porter lipoprotein | similar to laminin binding protein in iriae |  | Computational Prediction |
| **SSA_1457** | neopullulanase |  | dexB | Computational Prediction |
| **SSA_1492** | hypothetical protein |  | spxR | (Chen et al. 2012, Moraes et al. 2014) |
| **SSA_1505** | Hypothetical Protein | Partially similar to phosphoglycerol transferase-like proteins, alkaline phosphatase superfamily |  | Computational Prediction |
| **SSA_1509** | polysaccharide biosynthesis protein/ rhamnosyltransferase | cell wall polysaccharides…receptors for agglutination and coag in oral strep (ortholog in *S. gordonii*) | rgpB | (Xu et al. 2007) |
| **SSA_1510** | rhamnosyltransferase | cell wall polysaccharides…receptors for agglutination and coag in oral strep (ortholog in *S. gordonii*) | rgpA | (Xu et al. 2007) |
| **SSA_1511** | glycosyltransferase | cell wall polysaccharides…receptors for agglutination and coag in oral strep (ortholog in *S. gordonii*) |  | (Xu et al. 2007) |
| **SSA_1512** | hypothetical protein | cell wall polysaccharides…receptors for agglutination and coag in oral strep (ortholog in *S. gordonii*) |  | (Xu et al. 2007) |
| **SSA_1514** | cell-wall biogenesis glycosyltransferase | cell wall polysaccharides…receptors for agglutination and coag in oral strep (ortholog in *S. gordonii*) |  | (Xu et al. 2007) |
| **SSA_1515** | hypothetical protein | cell wall polysaccharides…receptors for agglutination and coag in oral strep (ortholog in *S. gordonii*) |  | (Xu et al. 2007) |
| **SSA_1516** | cell-wall biogenesis glycosyltransferase | cell wall polysaccharides…receptors for agglutination and coag in oral strep (ortholog in *S. gordonii*) |  | (Xu et al. 2007) |
| **SSA_1517** | cell-wall biogenesis glycosyltransferase | cell wall polysaccharides…receptors for agglutination and coag in oral strep (ortholog in *S. gordonii*) | cpslaJ | (Xu et al. 2007) |
| **SSA_1518** | glycosyl transferase | cell wall polysaccharides…receptors for agglutination and coag in oral strep (ortholog in *S. gordonii*) | rgpE | (Xu et al. 2007) |
| **SSA_1525** | Lyzozyme M1 (1,4-beta-N-acetylmuramidase) | similar to autolysin in iriae |  | Computational Prediction |
| **SSA_1541** | U32 family peptidase putative | similar to collagen binding in iriae |  | Computational Prediction |
| **SSA_1542** | U32 family peptidase putative | similar to collagen binding in iriae |  | Computational Prediction |
| **SSA_1546** | prolipoprotein diacylglyceryl transferase | LP processing | lgt | (Turner et al., 2009a; Xu et al., 2007) |
| **SSA_1564** | histidine kinase | VicRK component, biofilm reduction, eDNA reduction, downregulates cell envelope biogenesis (gbpB & 0094) |  | (Moraes et al. 2014) |
| **SSA_1576** | catabolite control protein A | represses spxB (0391) expression | ccpA | (Moraes et al. 2014) |
| **SSA_1631** | sortase-like protein | Pilin-associated sortase, colonization and virulence (ortholog in *S. pneumoniae*); CWA processing | srtC | (Xu et al. 2007) |
| **SSA_1632-1634** |  | Pilus locus |  | (Okahashi et al., 2010; 2011) |
| **SSA_1632** | surface protein | Pilus locus, colonization and virulence (ortholog in *S. pneumoniae*); LPXTG proteins, conserved "E box" |  | (Xu et al. 2007) |
| **SSA_1633** | FimA fimbrial subunit-like protein | Pilus locus, colonization and virulence (ortholog in *S. pneumoniae*); LPXTG proteins, conserved "E box" |  | (Xu et al. 2007, Herzberg 1996) |
| **SSA_1634** | Heme utilization/adhesion exoprotein | Pilus locus, colonization and virulence (ortholog in *S. pneumoniae*); LPXTG proteins, conserved "E box" |  | (Xu et al. 2007) |
| **SSA_1635** | hypothetical protein | Pilus locus, colonization and virulence (ortholog); LPXTG proteins |  | (Xu et al. 2007) |
| **SSA_1648** | Hypothetical Protein | cell envelope biogenesis |  | Computational Prediction |
| **SSA_1663** | collagen-binding protein A | Platelet aggregation, endocarditis virulence, collagen binding | cbpA | (Herzberg et al. 2005, Xu et al. 2007) |
| **SSA_1666** | collagen-binding surface protein | Collagen binding, pfam05737 | cbpB | (Herzberg et al. 2005, Xu et al. 2007) |
| **SSA_1761** | hemolysin | hemolysin (ortholog in *S. pneumoniae*) | hlyX | (Xu et al. 2007) |
| **SSA_1772** | Hypothetical Protein | Lipid metabolism? |  | Computational Prediction |
| **SSA_1882** | subtilisin-like serine proteases | C5A surface peptidase, virulence in mice (ortholog in *S. pyogenes*) | prtS | (Xu et al. 2007) |
| **SSA_1909** | transcriptional attenuator LytR | 60+% identical to biofilm regulatory protein A in mutans (homolog in *S. mutans*) |  | (Xu et al. 2007) |
| **SSA_1910** | Hypothetical Protein | conversion for coag receptor polysaccharides; similar to gene in gordonii that has this property |  | Computational Prediction |
| **SSA_1959** | undecaprenyl pyrophosphate phosphatase | endocarditis virulence | BacA | (Paik et al. 2005, Xu et al. 2007) |
| **SSA_1990** | Zn-porter lipoprotein | Laminin binding lipoprotein (ortholog in *S. agalactiae*) |  | (Turner et al. 2009a, Xu et al. 2007) |
| **SSA_1991** | pneumococcal histidine triad protein A | Surface C3 protease (ortholog in *S. pneumoniae*) | phtA | (Xu et al. 2007) |
| **SSA_2019** | Hypothetical Protein | signal transduction, ACT domain-containing protein |  | Computational Prediction |
| **SSA_2118** | thiamine pyrophosphokinase |  | tpk | (Chen et al. 2012, Moraes et al. 2014) |
| **SSA_2173** | aminoacylase/N-acyl-L-amino acid amidohydrolase/hippurate hydrolase | similar to autolysin in iriae | hipO | Computational Prediction |
| **SSA_2188** | Hypothetical Protein | inorganic ion transport |  | Computational Prediction |
| **SSA_2209** | penicillin-binding protein 2A | operon with 2211? | pbp2a | Computational Prediction |
| **SSA_2212** | polysaccharide transport protein | cell wall polysaccharides…receptors for agglutination and coag in oral strep (ortholog in *S. gordonii*) |  | (Xu et al. 2007) |
| **SSA_2213** | nucleotide sugar dehydratase | cell wall polysaccharides…receptors for agglutination and coag in oral strep (ortholog in *S. gordonii*) |  | (Xu et al. 2007) |
| **SSA_2214** | 2-C-methyl-D-erythritol 4-phosphate cytidylyltransferase 2 | cell wall polysaccharides…receptors for agglutination and coag in oral strep (ortholog in *S. gordonii*) |  | (Xu et al. 2007) |
| **SSA_2215** | oligosaccharide repeat-containing polymerase | cell wall polysaccharides…receptors for agglutination and coag in oral strep (ortholog in *S. gordonii*) |  | (Xu et al. 2007) |
| **SSA_2216** | LPS biosynthesis protein | cell wall polysaccharides…receptors for agglutination and coag in oral strep (ortholog in *S. gordonii*) | licD1 | (Xu et al. 2007) |
| **SSA_2217** | Cps9H | cell wall polysaccharides…receptors for agglutination and coag in oral strep (ortholog in *S. gordonii*) |  | (Xu et al. 2007) |
| **SSA_2218** | glycosyltransferase (cell wall biogenesis) Cps9G | cell wall polysaccharides…receptors for agglutination and coag in oral strep (ortholog in *S. gordonii*) |  | (Xu et al. 2007) |
| **SSA_2219** | UDP-glucose 4-epimerase | cell wall polysaccharides…receptors for agglutination and coag in oral strep (ortholog in *S. gordonii*) |  | (Xu et al. 2007) |
| **SSA_2220** | galactosyltransferase | cell wall polysaccharides…receptors for agglutination and coag in oral strep (ortholog in *S. gordonii*) |  | (Xu et al. 2007) |
| **SSA_2221** | Cps9E | cell wall polysaccharides…receptors for agglutination and coag in oral strep (ortholog in *S. gordonii*) | capD | (Xu et al. 2007) |
| **SSA_2222** | tyrosine-protein kinase Wze | cell wall polysaccharides…receptors for agglutination and coag in oral strep (ortholog in *S. gordonii*) |  | (Xu et al. 2007) |
| **SSA_2223** | capsular polysaccharide biosynthesis protein Wzd (chain length regulator) | cell wall polysaccharides…receptors for agglutination and coag in oral strep (ortholog in *S. gordonii*) |  | (Xu et al. 2007) |
| **SSA_2224** | phosphotyrosine-protein phosphatase | cell wall polysaccharides…receptors for agglutination and coag in oral strep (ortholog in *S. gordonii*) |  | (Xu et al. 2007) |
| **SSA_2225** | transcriptional attenuator LytR | cell wall polysaccharides…receptors for agglutination and coag in oral strep (ortholog in *S. gordonii*) | cspA | (Xu et al. 2007) |
| **SSA_2230** | anaerobic ribonucleoside triphosphate reductase | endocarditis virulence | nrdD | (Paik et al. 2005, Rhodes et al. 2014, Xu et al. 2007) |
| **SSA_2300** | Hypothetical Protein | inorganic ion transport |  | Computational Prediction |
| **SSA_2302** | Type IV fimbrial biogenesis protein, prepilin cysteine protease (C20) PilD | homology to T4 pili |  | (Xu et al. 2007) |
| **SSA_2333** | Hypothetical Protein | cell envelope biogenesis | dltB | Computational Prediction |
| **SSA_2338** | Hypothetical Protein | similar to IgA1 Protease |  | Computational Prediction |
| **SSA_2364** | Hypothetical Protein | cell envelope biogenesis |  | Computational Prediction |

**References**

Black, C., Allan, I., Ford, S. K., Wilson, M., and McNab, R. (2004). Biofilm-specific surface properties and protein expression in oral Streptococcus sanguis. *Archives of Oral Biology* 49, 295–304. doi:10.1016/j.archoralbio.2003.12.001.

Chen, L., Ge, X., Wang, X., Patel, J. R., and Xu, P. (2012). SpxA1 Involved in Hydrogen Peroxide Production, Stress Tolerance and Endocarditis Virulence in Streptococcus sanguinis. *PLoS ONE* 7, e40034. doi:10.1371/journal.pone.0040034.

Das, S., Kanamoto, T., Ge, X., Xu, P., Unoki, T., Munro, C. L., et al. (2009). Contribution of Lipoproteins and Lipoprotein Processing to Endocarditis Virulence in Streptococcus sanguinis. *Journal of Bacteriology* 191, 4166–4179. doi:10.1128/jb.01739-08.

Fan, J., Zhang, Y., Chuang-Smith, O. N., Frank, K. L., Guenther, B. D., Kern, M., et al. (2012). Ecto-5′-Nucleotidase: A Candidate Virulence Factor in Streptococcus sanguinis Experimental Endocarditis. *PLoS ONE* 7, e38059. doi:10.1371/journal.pone.0038059.

Ge, X., Kitten, T., Chen, Z., Lee, S. P., Munro, C. L., and Xu, P. (2008). Identification of Streptococcus sanguinis Genes Required for Biofilm Formation and Examination of Their Role in Endocarditis Virulence. *Infection and Immunity* 76, 2551–2559. doi:10.1128/IAI.00338-08.

Ge, X., Yu, Y., Zhang, M., Chen, L., Chen, W., Elrami, F., et al. (2016). Involvement of NADH Oxidase in Competition and Endocarditis Virulence in Streptococcus sanguinis. *Infection and Immunity* 84, 1470–1477. doi:10.1128/IAI.01203-15.

Herzberg, M. C. (1996). Platelet-Streptococcal Interactions in Endocarditis. *Critical Reviews in Oral Biology & Medicine* 7, 222–236. doi:10.1177/10454411960070030201.

Herzberg, M. C., Nobbs, A., Tao, L., Kilic, A., Beckman, E., Khammanivong, A., et al. (2005). Oral Streptococci and Cardiovascular Disease: Searching for the Platelet Aggregation-Associated Protein Gene and Mechanisms of Streptococcus sanguis-Induced Thrombosis. *Journal of Periodontology* 76, 2101–2105. doi:10.1902/jop.2005.76.11-S.2101.

Moraes, J. J., Stipp, R. N., Harth-Chu, E. N., Camargo, T. M., Höfling, J. F., and Mattos-Graner, R. O. (2014). Two-Component System VicRK Regulates Functions Associated with Establishment of Streptococcus sanguinis in Biofilms. *Infection and Immunity* 82, 4941–4951. doi:10.1128/IAI.01850-14.

Okahashi, N., Nakata, M., Sakurai, A., Terao, Y., Hoshino, T., Yamaguchi, M., et al. (2010). Pili of oral Streptococcus sanguinis bind to fibronectin and contribute to cell adhesion. *Biochemical and Biophysical Research Communications* 391, 1192–1196. doi:10.1016/j.bbrc.2009.12.029.

Okahashi, N., Nakata, M., Terao, Y., Isoda, R., Sakurai, A., Sumitomo, T., et al. (2011). Pili of oral Streptococcus sanguinis bind to salivary amylase and promote the biofilm formation. *Microbial Pathogenesis* 50, 148–154. doi:10.1016/j.micpath.2011.01.005.

Paik, S., Senty, L., Das, S., Noe, J. C., Munro, C. L., and Kitten, T. (2005). Identification of virulence determinants for endocarditis in Streptococcus sanguinis by signature-tagged mutagenesis. *Infection and Immunity* 73, 6064–74. doi:10.1128/IAI.73.9.6064-6074.2005.

Turner, L., Kanamoto, T., Unoki, T., Munro, C. L., Wu, H., and Kitten, T. (2009a). Comprehensive Evaluation of Streptococcus sanguinis Cell Wall-Anchored Proteins in Early Infective Endocarditis. *Infection and Immunity* 77, 4966–4975. doi:10.1128/IAI.00760-09.

Turner, S. L., Das, S., Kanamoto, T., Munro, C., and Kitten, T. (2009b). Development of genetic tools for in vivo virulence analysis of Streptococcus sanguinis. *Microbiology* 155, 2573–2582. doi:10.1099/mic.0.024513-0.

Xu, P., Alves, J., Kitten, T., Brown, A., and Chen, Z. (2007). Genome of the opportunistic pathogen Streptococcus sanguinis. *Journal of Bacteriology* 189, 3166–3175. doi:10.1128/JB.01808-06.
